# Genomic Distribution of *ushA*-like Genes in *Bacteria*: Comparison to *cpdB*-like Genes

**DOI:** 10.3390/genes14081657

**Published:** 2023-08-20

**Authors:** João Meireles Ribeiro, José Carlos Cameselle

**Affiliations:** Grupo de Enzimología, Departamento de Bioquímica y Biología Molecular y Genética, Facultad de Medicina y Ciencias de la Salud, Universidad de Extremadura, 06006 Badajoz, Spain; camselle@unex.es

**Keywords:** UshA, CpdB, nucleotidase, bacteria, genome, TBlastN

## Abstract

UshA and CpdB are nucleotidases of the periplasm of several Gram-negative bacteria, while several Gram-positives contain cell wall-bound variants. UshA is a 5′-nucleotidase, a UDP-sugar hydrolase, and a CDP-alcohol hydrolase. CpdB acts as a 3′-nucleotidase and as a phosphodiesterase of 2′,3′-cyclic nucleotides and 3′,5′-linear and cyclic dinucleotides. Both proteins are pro-virulent for the pathogens producing them and facilitate escape from the innate immunity of the infected host. Recently, the genomic distribution of *cpdB*-like genes in *Bacteria* was found to be non-homogeneous among different taxa, and differences occur within single taxa, even at species level. Similitudes and differences between UshA-like and CpdB-like proteins prompted parallel analysis of their genomic distributions in *Bacteria*. The presence of *ushA*-like and *cpdB*-like genes was tested by TBlastN analysis using seven protein probes to query the NCBI Complete Genomes Database. It is concluded that the distribution of *ushA*-like genes, like that of *cpdB*-like genes, is non-homogeneous. There is a partial correlation between both gene kinds: in some taxa, both are present or absent, while in others, only one is present. The result is an extensive catalog of the genomic distribution of these genes at different levels, from phylum to species, constituting a starting point for research using other in silico or experimental approaches.

## 1. Introduction

The proteins UshA and CpdB are prototypic nucleotidases of the periplasmic space of *Escherichia coli* [1,2] and other Gram-negative bacteria [3,4,5,6,7,8,9,10]. As far as enzyme activity is concerned, UshA is a highly efficient 5′-nucleotidase that is also active as a phosphoanhydride hydrolase of UDP-sugars, CDP-alcohols, and other nucleotidic derivatives [11,12,13]. CpdB is a highly efficient 3′-nucleotidase, also active as a phosphodiesterase of 2′,3′-cyclic mononucleotides, 3′,5′-cyclic or linear dinucleotides, and the artificial phosphodiester substrate bis-4-nitrophenylphosphate [14,15]. Both proteins are structurally related, as following the removable signal peptide for secretion (SP), they display the same two-domain architecture: an N-terminal metallophos domain (Pfam ID PF00149) that includes the catalytic site with a dimetal center, and a C-terminal 5_nucleotid_C domain (Pfam ID PF02872) that includes a substrate-binding site [16,17]. It is noteworthy that the designation of the 5_nucleotid_C domain does not imply the occurrence of 5′-nucleotidase activity. UshA is a 5′-nucleotidase devoid of 3′-nucleotidase activity, while CpdB is a 3′-nucleotidase devoid of 5′-nucleotidase activity. In both proteins, the N- and C-terminal domains are joined by a ≈20-amino acid linker [17,18]. Both enzymes are believed to share a remarkable catalytic cycle in which the typical 5′-AMP or 3′-AMP substrates bind to the specificity site in the 5_nucleotid_C domain, with the adenine ring forming a stacking sandwich between two aromatic residues. The substrate-charged domains then undergo large, 96° rotations that bring the substrate to the catalytic site in the metallophos domains, where dephosphorylation takes place [17,19,20].

Besides the periplasmic versions of UshA and CpdB, UshA-like and CpdB-like proteins occur in Gram-positive bacteria as cell wall-bound proteins that have received different names. Among them are ecto-5′-nucleotidases of *Staphylococcus aureus* (AdsA [21]), *Streptococcus sanguinis* (Nt5e [22]), *S. agalactiae* (NudP [23]), *S. pyogenes* (S5nA [24]), and *S. suis* (Ssads [25]), and ecto-3′-nucleotidases of *S. agalactiae* (CdnP [26]) and *S. suis* (SntA [27,28]). These proteins, in addition to the SP, metallophos, and 5_nucleotid_C domains typical of UshA and CpdB, bear 3′ extensions that constitute cell wall binding domains with a LPXTG or similar motif [29].

The periplasmic or cell wall locations of these enzymes make them able to act on non-cytoplasmic substrates, either secreted from the same cell or of exogenous origin, for instance, in the cytoplasm of eukaryotic cells invaded by bacterial pathogens such as *Salmonella enterica* or *S. agalactiae*. Both 5′-nucleotidases and 3′-nucleotidases have been identified and considered virulence factors for producing pathogens by mechanisms related to their nucleotide-degrading activities or to effects on complement that facilitate evasion from host innate immunity [21,22,23,24,25,26,27,30,31].

For these reasons, we consider it of utmost interest to gain knowledge of how widespread, among the genomes of different bacterial taxa, the occurrence of genes coding for nucleotidases is, which, either by being periplasmic or bound to the cell wall, have the potential to act extracytoplasmatically on nucleotidic substrates. In a recent study, we analyzed the genomic distribution of *cpdB*-like genes using the protein sequence of *S. enterica* CpdB as a probe (query) for TBlastN analyses of complete genomes, limited by bacterial taxa at different levels, from phyla to species [32]. The results revealed that *cpdB*-like genes are far from ubiquitous in the superkingdom *Bacteria*, being present in some phyla but not in others. At levels higher than species, the genomic distribution was not homogeneous since few taxa contained a *cpdB*-like gene in all the sequenced genomes. At the level of species, the distribution was more homogeneous, as out of 77 taxa considered, 38 showed a (near) widespread distribution of *cpdB*-like genes and 28 did not contain them. Interestingly, 11 species showed a partial distribution, with some sequenced genomes but not all containing a *cpdB*-like gene. This interesting panoramic view prompted us to extend the analysis to *ushA*-like genes and to perform it in a more detailed way by increasing the number of TBlastN probes from the single one used in the previous study [32] to a total of seven different probes in the current manuscript, five for *usha*-like genes and two for *cpdB*-like ones. The result is an extensive catalog of the genomic distribution of these genes at different levels, from phylum to species, constituting a starting point for research using other in silico or experimental approaches. Major observations were that the genomic distribution of *ushA*-like genes was not homogeneous and that the correlation with *cpdB*-like genes was partial, as in some taxa both were present or absent; however, in others only one was present. Other interesting outcomes worth further research by other approaches are pointed out.

## 2. Materials and Methods

**Figure 1:**
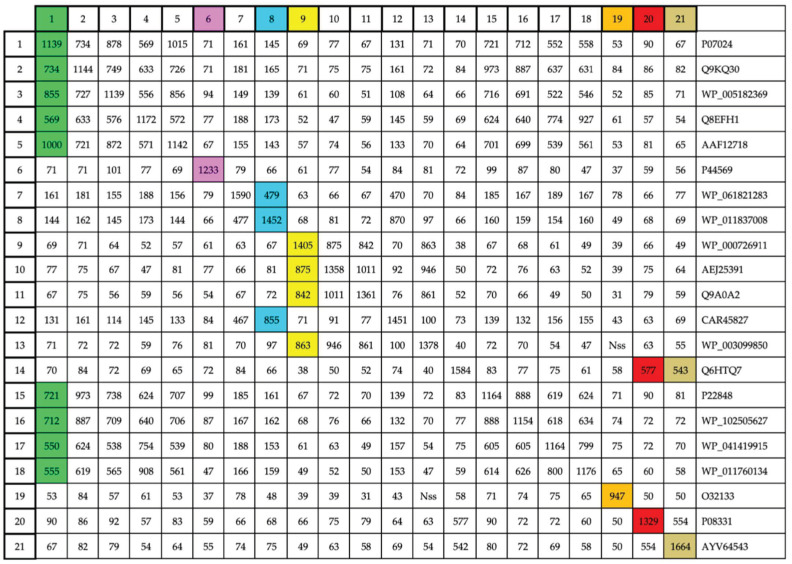
Mutual relatedness among the set of proteins used to select TBlastN probes for bacterial genome analysis. An internal BlastP comparison was run among the 21 proteins in Table 1. The grid intersections show the alignment scores obtained. On the top line, the seven proteins selected for use are colored as in Table 1 to facilitate cross-referencing. Within the grid, the colors identify the proteins with high scores, indicative of strong relatedness. The seven probes selected cover, with high scores, the whole set of proteins. Nss, not significant similitude.

TBlastN analyses [33,34] were run against NCBI complete microbial genomes (https://blast.ncbi.nlm.nih.gov/Blast.cgi?PROGRAM=TBlastN&PAGE_TYPE=BlastSearch&BLAST_SPEC=MicrobialGenomes&LINK_LOC=blasttab&LAST_PAGE=blastn, accessed on 15 July 2023). Default parameters were applied except that the maximum number of target sequences was adapted to the expected number of hits. The database was queried using the sequence identifiers of the seven-probe set selected (Figure 1). Routinely, the Entrez query “NOT plasmid [Title]” was applied. The searches within each taxonomical group (taxid) (Organism) were restricted in principle to genomes of type material [35]. This restriction was removed when less than five type-material genomes were available or, as a rule, for searches within genera and species. The typical conditions for launching a TBlastN search from the Microbial Translated Blast page are shown in Figure S1. When running searches limited by organism, a bug was observed in the organism menu as it occasionally chose the wrong taxid number. Therefore, all taxid numbers were checked in the NCBI Taxonomy browser (https://www.ncbi.nlm.nih.gov/Taxonomy/Browser/wwwtax.cgi, acceessed on 15 July 2023) [36]. Genomic hits were computed when the alignment score was >150 and query coverage was >70%.

## 3. Results

### 3.1. Selection of Probes for TBlastN Analysis

The probes for TBlastN analysis of UshA-like genes were selected among a set of 21 bacterial 5′-nucleotidases (Table 1). Eighteen of them were collected from a recent review by Zakataeva [37], to which two well-characterized CpdB-like 3′-nucleotidases were added [14,27] (no. 20 and 21 in Table 1). All of them are either periplasmic or cell wall-bound, experimentally studied nucleotidases. In addition, one uncharacterized, putative 5′-nucleotidase of *B. subtilis*, recovered from UniProtKB/Swiss-Prot (https://www.uniprot.org/help/uniprotkb, acceessed on 15 July 2023) [38], was included (no. 19 in Table 1).

**Table 1:** Periplasmic or cell wall-bound bacterial nucleotidases are used to select probes for TBlastN analysis of bacterial genomes. This protein set was taken from Zakataeva [37], except no. 19 (taken from UniProtKB/Swiss-Prot [38]) and no. 20 and 21 from [14,27].

No.	Accession	Description	Amino Acids
1 ^1^	P07024	USHA_ECOLI [*Escherichia coli*]	550
2	Q9KQ30	5′-nucleotidase [*Vibrio cholerae*]	553
3	WP_005182369	UDP-sugar hydrolase/5′-nucleotidase UshA [*Yersinia intermedia*]	550
4	Q8EFH1	5′-nucleotidase [*Shewanella oneidensis*]	569
5	AAF12718	UDP-sugar hydrolase precursor [*Klebsiella aerogenes*]	550
6	P44569	NAD 5′-nucleotidase [*Haemophilus influenzae*]	603
7	WP_061821283	LPXTG-anchored adenosine synthase AdsA [*Staphylococcus aureus*]	772
8	WP_011837008	Cell surface ecto-5′-nucleotidase Nt5e [*Streptococcus sanguinis*]	719
9	WP_000726911	Bifunctional metallophosphatase/5′-nucleotidase [*Streptococcus agalactiae*]	690
10	AEJ25391	Surface-anchored 5′-nucleotidase [*Streptococcus equi*]	668
11	Q9A0A2	Putative secreted 5′-nucleotidase (5′-nucleotidase) [*Streptococcus pyogenes*]	670
12	CAR45827	Putative 5′-nucleotidase [*Streptococcus suis* P1/7]	721
13	WP_003099850	5′-Nucleotidase C-terminal domain-containing protein [*Streptococcus iniae*]	676
14	Q6HTQ7	2′,3′-Cyclic-nucleotide 2′-phosphodiesterase [*Bacillus anthracis*]	780
15	P22848	5′-Nucleotidase (*Vibrio parahaemolyticus*)	560
16	WP_102505627	UDP-sugar hydrolase/5′-nucleotidase UshA [*Salinivibrio costicola*)	557
17	WP_041419915	UDP-sugar hydrolase/5′-nucleotidase UshA [*Shewanella violacea*]	569
18	WP_011760134	UDP-sugar hydrolase/5′-nucleotidase UshA [*Shewanella amazonensis*]	571
19	O32133	Uncharacterized metallophosphoesterase YunD [*Bacillus subtilis*]	462
20	P08331	2′,3′-Cyclic-nucleotide 2′-phosphodiesterase/3′-nucleotidase [*Escherichia coli*]	647
21	AYV64543	Heme-binding protein SntA [*Streptococcus suis*]	813

^1^ The probes selected for the study are shown in the background color as Figure 1 to facilitate cross-referencing.

The mutual relatedness among Table 1 proteins was evaluated by the scores of BlastP alignments (Figure 1). This allowed us to select seven proteins to be used as TBlastN probes; they are identified as proteins 1, 6, 8, 9, 19, 20, and 21 in Table 1 and Figure 1. According to the color code used in Figure 1, five of the selected probes (no. 1, 8, 9, 20, and 21) were highly related to a small group of nucleotidases, whereas the other two probes (no. 6 and 19) showed insignificant alignment scores with any other member of the set. Incidentally, one of the so-called 5′-nucleotidases (no. 14 in Table 1) was actually a CpdB-like protein, to judge from its strong relatedness to authentic CpdB-like enzymes (no. 20 and 21 in Table 1) and insignificant alignment scores to the other Table 1 proteins.

### 3.2. TBlastN Analysis of Bacterial Genomes

#### 3.2.1. General Strategy for the Analysis and Presentation of Results

TBlastN searches were run between 30 June and 15 July 2023 as described under the Materials and Methods section. The number of hits obtained with each probe for each taxonomical group analyzed was recorded. A global TBlastN search was run on 30 June 2023, in the superkingdom *Bacteria* (taxid:2), with 4185 type-material genomes and a total of 40,608 genomes available on that date. Table 2 shows the results obtained with the seven probes, computing only hits found among type-material genomes.

Thereafter, searches were run in the *Bacteria* taxa of the NCBI Taxonomy browser [36] at different levels. Detailed results are shown in Tables S1–S6. Summaries of the results at different levels are shown in Table 3, Table 4, Table 5, Table 6, Table 7 and Table 8, where the presence or absence of *ushA*-like and *cpdB*-like genes is schematically indicated. In these summaries, “presence” does not mean that the genes are widespread in the taxon. In this concern, three levels are distinguished: ≤50%, >50% but <100%, and 100% of the analyzed genomes contain *ushA*-like and/or *cpdB*-like genes (hits). This is marked by the letters U (*ushA*-like) and C (*cpdB*-like) on different color backgrounds: green (≤50%), orange (>50% but <100%), and red (100%). It must be remarked that these percentages include hits obtained with any of the UshA-like or CpdB-like probes (see Table 2). For *ushA*-like genes, the results were strongly dependent on the probe (five different ones are used), whereas for *cpdB*-like genes, the two probes used gave similar results in most but not all the taxonomical groups. Finally, when “presence” of both types of genes is indicated for the same taxon, it does not necessarily mean they are in the same genome, unless 100% of the genomes gave positive results in both cases. Of course, 100% positivity for one gene type and <100% for the other means that some genomes contain both types of genes. On the other hand, logically, “absence” refers to 100% of the analyzed genomes with any probe.

In summary, when a taxonomical level is negative or 100% positive for both genes, or negative for one and 100% positive for the other gene type, the analysis of such a taxon is deemed complete and is not pursued further at lower taxonomical levels.

#### 3.2.2. Genomic Distribution of *ushA*-like and *cpdB*-like Genes in *Bacteria* Phyla

Forty three phyla, including the Delta/epsilon subdivision, found in the NCBI Taxonomy browser within the superkingdom *Bacteria* (mostly coincident with [39]), were submitted to TBlastN analyses with UshA-like and CpdB-like probes (Table 2). The detailed results are shown in Table S1. A simpler summary of the presence/absence of *ushA*-like and *cpdB*-like genes is shown in Table 3.

Twelve phyla contained both types of genes; 14 showed neither; 11 showed *ushA*-like but not *cpdB*-like genes; and in six cases, the converse was true. With the criteria of Section 3.2.1, 19 phyla (those in black type in Table 3) were considered complete and not pursued at lower levels. The 24 phyla in red type are further analyzed in Table 4 and Table S2.

#### 3.2.3. Genomic Distribution of *ushA*-like and *cpdB*-like Genes in Bacterial Classes of Selected Phyla

To continue the TBlastN exploration, 76 bacterial classes belonging to 24 different phyla were queried with the seven probes (Table 2). The detailed results are shown in Table S2. In six of those classes, there was no sequenced genome. A simpler summary of the presence/absence of *ushA*-like and *cpdB*-like genes in the 70 classes for which there were sequenced genome(s) is shown in Table 4. With the criteria defined in Section 3.2.1, the analyses of 31 classes (those in black type in Table 4) were considered complete and not pursued further at lower taxonomical levels. On the other hand, the 39 classes shown in red type in Table 4 were further analyzed (Table 5 and Table S3), with one exception marked with an asterisk.

#### 3.2.4. Genomic Distribution of *ushA*-like and *cpdB*-like Genes in Bacterial Orders of Selected Classes

To continue the TBlastN exploration, 152 bacterial orders belonging to 38 different classes were queried with the seven probes (Table 2). The detailed results are shown in Table S3. In 20 of those classes, there was no sequenced genome. A simpler summary of the presence/absence of *ushA*-like and *cpdB*-like genes in the 132 classes for which there were sequenced genome(s) is shown in Table 5. With the criteria defined in Section 3.2.1, the analyses of 53 orders (those in black type in Table 5) were considered complete and not pursued further at lower taxonomical levels. On the other hand, the 79 orders shown in red type in Table 5 were further analyzed (Table 6 and Table S4).

#### 3.2.5. Genomic Distribution of *ushA*-like and *cpdB*-like Genes in Bacterial Families of Selected Orders

To continue the TBlastN exploration, 403 bacterial families belonging to 79 different orders were queried with the seven probes (Table 2). The detailed results are shown in Table S4. In 99 of those families, there was no sequenced genome. A simpler summary of the presence/absence of *ushA*-like and *cpdB*-like genes in the 304 families for which there were sequenced genome(s) is shown in Table 6. With the criteria defined in Section 3.2.1, the analyses of 139 families (those in black type in Table 6) were considered complete and not pursued further at lower levels. On the other hand, the 165 families shown in red type in Table 6 were further analyzed (Table 7 and Table S5).

#### 3.2.6. Genomic Distribution of *ushA*-like and *cpdB*-like Genes in Bacterial Genera of Selected Families

To continue the TBlastN exploration, 510 bacterial genera belonging to 165 different families were queried with the seven probes (Table 2). In contrast to previous steps (Section 3.2.3, Section 3.2.4 and Section 3.2.5), TBlastN analyses were not run for all the genera belonging to the families deemed not complete (those highlighted in red type in Table 6). Instead, while doing the TBlastN analyses of families, the genera giving the hits were annotated, thus avoiding running later lots of TBlastN searches that would not give any hits.

The detailed results obtained with the 510 selected genera are shown in Table S5. For all of them, the NCBI Complete Genomes Database contained at least one sequenced genome. A simpler summary of the presence/absence of *ushA*-like and *cpdB*-like genes in those genera is shown in Table 7.

With the criteria defined in Section 3.2.1, the analyses of 268 genera (those in black type in Table 7) were considered complete. Anyhow, for analyses at the level of species (Table 8 and Table S6), also at variance with previous steps, the selection was not based on the non-complete character of the genera. Instead, the selection was purely subjective and included species belonging to genera not mentioned in Table 7, as explained in Section 3.2.7.

#### 3.2.7. Genomic Distribution of *ushA*-like and *cpdB*-like Genes in Selected Bacterial Species 

To continue the TBlastN exploration, 107 bacterial species belonging to different families were queried with the seven probes (Table 2). In contrast to the strategy followed at the previous taxonomical levels, when systematic criteria were applied for taxa selection (Section 3.2.3, Section 3.2.4, Section 3.2.5 and Section 3.2.6), a subjective selection of species was made in this case. It included all the bacterial species analyzed in the earlier study of *cpdB*-like genes, which had been selected mainly for their pathogenicity [32]. In summary, 107 different species were queried with the seven probes. Of them, the 80 shown in black type were declared complete by the criteria described in Section 3.2.1. Non-complete species are highlighted in red. Detailed results are in Table S6, and a summary is in Table 8.

## 4. Discussion

### 4.1. Overview

This study is a follow-up of a previous analysis of the genomic distribution of *cpdB*-like genes in *Bacteria*, which was performed with *S. enterica* CpdB (GenBank accession P26265) as the probe [32]. That study was mainly centered on the phyla *Pseudomonadota* and *Bacillota* (named then more traditionally as *Proteobacteria* and *Firmicutes*, respectively) and their lower divisions. In the current manuscript, the former study has been extended in several aspects, mainly that besides *cpdB*-like genes, *ushA*-like genes have been analyzed, and the searches were run without a priori restriction to particular taxa. Moreover, several probes were used, two for *cpdB*-like genes and five for *ushA*-like genes (Table 2). The use of several UshA-like probes revealed different types of *ushA*-like genes, some of them specifically associated with different bacterial taxa. The result is an extensive catalog of the distribution of these genes in superkingdom *Bacteria*. Several resources are provided, including Supplementary Tables S1–S6 that contain the detailed results of the analyses at different levels: phylum (Table S1), class (Table S2), order (Table S3), family (Table S4), genus (Table S5), and species (Table S6). In the main manuscript, Table 3, Table 4, Table 5, Table 6, Table 7 and Table 8 contain summaries of the data at different levels, from phylum to species.

Table 9 summarizes the total numbers of taxa studied, including the counts of probed taxa of different levels, analyzed taxa (once discounted those for which, by the time of submission, upon TBlastN, no sequenced genomes were found in the NCBI Complete Genomes Database), and taxa declared complete according to the criteria explained in Section 3.2.1. For complete taxa, Table 9 also shows the breakdown by kind of results, depending on whether UshA-like and/or CpdB-like probes gave hits or not. To facilitate searching for particular taxa, alphabetical lists are provided of the 1291 taxa probed (Table S7) and of the 125 taxa without sequenced genomes in the NCBI Complete Genomes Database among those that were probed (Table S8).

### 4.2. About the Possible Correlation between ushA-like and cpdB-like Genes

UshA and CpdB have different specificities. UshA is a 5′-nucleotidase, UDP-sugar hydrolase, and CDP-alcohol hydrolase [11,12], and CpdB acts as a 3′-nucleotidase and as a phosphodiesterase of 2′,3′-cyclic nucleotides and 3′,5′-linear and cyclic dinucleotides [14]. They are periplasmic [1,2] or cell-wall [21,22,23,24,25,26,27] enzymes that act on extracellular substrates, either exogenous or endogenous. In addition, both are provirulent factors for the producing pathogens, facilitating escape from the innate immunity of the host [21,22,23,24,25,26,27]. The similitude between them was the main reason to study and compare their genomic distributions in *Bacteria*, with the aim of establishing the extent to which the occurrence of one correlates with the occurrence of the other. In this regard, it is worth recalling that, for instance, the action of CpdB-like proteins on linear and cyclic dinucleotides yields 5′-nucleotides as products but cannot continue their degradation to nucleosides [32]. To this end, the metabolic action of CpdB-like enzymes can be continued by UshA-like enzymes. Moreover, pointing to the correlation between both enzymes is the occurrence in some bacteria of natural fusions of UshA and CpdB as the result of two-gene fusion [40,41].

Table 10, Table 11, Table 12 and Table 13 summarize the non-homogeneous distribution of both gene kinds and the (lack of) correlation between them. A qualitative correlation was observed between both gene kinds for some taxa but not for others. In 416 out of 590 taxa (70.5%), they were both either present (31.4%; Table 10) or absent (39.1%; Table 11). However, 174 taxa (29.5%) failed to show such a correlation, as one of the gene types was present but not the other: 21.7% of the taxa bear *ushA*-like, not *cpdB*-like genes (Table 12), whereas for 7.8% the converse was true (Table 13).

### 4.3. Comments on Some Specific Stories

#### 4.3.1. About Phylum *Bacillota* and Class *Bacilli*: Specificity of Probe O32133

A phylum worth special attention is *Bacillota*, as half of the sequenced type-material genomes (366/705) gave hits with probe O32133, which gave no hits in other phyla except four hits found when the limit to type material was removed for the TBlastN. In *Bacillota,* there were many hits with all the UshA-like and CpdB-like probes (Table S1). In Table 14, *Bacillota* is compared with the rest of the superkingdom *Bacteria*.

In Table 15, a similar comparison is made between the class *Bacilli* and the rest of the phylum *Bacillota.* In every case, the distribution of hits among the sequenced genomes was partial, i.e., there were genomes with *ushA*-like and *cpdB*-like genes and genomes without them. The degree of coincidence cannot be easily ascertained at this level. This must be attempted at lower taxonomical levels.

#### 4.3.2. About the Occurrence of *ushA*-like and *cpdB*-like Genes in the Numerous Sequenced Genomes of *Escherichia coli*, *Salmonella enterica*, *Pasteurella multocida*, *Klebsiella pneumoniae* and *Vibrio cholerae*

The five species to be discussed in this section have in common that for them there are numerous genomes available in the NCBI Complete Genomes Database and that the TBlastN analyses did not give complete results for any of the two kinds of genes according to the criteria described in Section 3.2.1.

When complete results are obtained at least for one gene kind, e.g., *ushA*-like genes, the full picture can be inferred: there will be a number of genomes with both kinds present, and the remaining, up to the total number of genomes, will display only the *ushA* kind, or vice versa. This is the case for several of the species analyzed in Table 8 (with more details in Table S6). For instance, for *Staphylococcus saprophyticus,* there are 17 genomes available in the database; all of them gave a *ushA*-like hit, whereas only four gave a *cpdB*-like one (Table S6, line 92). It can be concluded that four genomes contain both gene kinds, and the remaining 13 contain only an *ushA*-like one.

In the cases to be discussed below, there were many hits with UshA-like and CpdB-like probes; however, since TBlastN did not give complete results in any case, for some genomes, it was unclear whether both kinds of genes were absent or one was present and the other absent.

The most important part of the structural and functional information of UshA and CpdB nucleotidases and their encoding genes has been obtained in *E. coli* [1,2,11,12,14,16,17,18,19]. For this species, a large number of genomes are available in the Complete Genomes Database (3565 when this manuscript was submitted). Most of them; however, not all contain both *ushA*-like and *cpdB*-like genes. According to data in line 35 of Table S6, there are 20 *E. coli* genomes that do not contain an *ushA*-like gene and 10 genomes that do not contain a *cpdB*-like gene. By downloading the TBlastN results obtained for *E. coli* with probes P07024 (UshA protein) and P08331 (CpdB protein), it was confirmed that the same 3559 *E. coli* genomes had been hit in both cases. Based on their alignment scores, it was possible to identify four genomes that contain an *ushA*-like gene but are devoid of a *cpdB*-like gene and 14 genomes for which the converse is true (Table 16). These exceptions were found in 10 different *E. coli* strains. In addition, there may be six non-identified genomes that contain neither *ushA*-like nor *cpdB*-like genes. These data confirm that, although *E. coli* is a major contributor to the occurrence of these genes in *Bacteria*, their distribution is near but not fully homogeneous, and there is a high but not full correlation between them.

A particular aspect of some *E. coli* genomes worthy of attention refers to those of the avian pathogenic *E. coli* (APEC) strains, for which the *cpdB* gene has been shown to be provirulent [30]. In the complete genome database, there are five APEC genomes, all of them containing *ushA* and *cpdB* genes with high scores (Table 17).

In contrast to what was found in *E. coli*, a similar analysis with *S. enterica* genomes led to different results. Table S6 (lines 79–80) shows data for two well-known variants of *S. enterica* subspecies enterica, serovars Typhi and Typhimurium. In the first case, the 125 genomes available for serovar Typhi gave all high-score hits with probes P07024 (UshA protein) and P08331 (CpdB protein). However, the 350 genomes available for serovar Typhimurium gave hits in only 219 cases with both probes. Downloading and inspection of TBlastN results indicated that all these genomes contained both *ushA*-like and *cpdB*-like genes, whereas the remaining 131 genomes of *Salmonella* Typhimurium deposited in the NCBI Complete Genomes Database contain none of them. In this case, no genome was identified containing one of the gene types but not the other. Therefore, the distribution of these genes in *Salmonella* Typhimurium is clearly not homogeneous; however, there is a good correlation between them.

In the case of *P. multocida*, 138 genomes were available in the database, of which 135 gave *ushA*-like hits and 137 gave *cpdB*-like ones (line 66 of Table S6). By downloading the TBlastN results obtained for this species with probes P07024 (UshA protein) and P08331 (CpdB protein), it was possible to identify one genome that, despite containing a high-score *ushA*-like gene, was devoid of a *cpdB*-like gene, and four genomes that, despite showing high-score *cpdB*-like genes, were devoid of *ushA*-like ones (or in one case, just a borderline hit) (Table 18). In this case, such as in *E. coli*, the distributions of both genes are near but not fully homogeneous, and there is a high but not full correlation between them.

*K. pneumoniae* is another species for which a large number of genomes are available in the Complete Genomes Database (1967, when this manuscript was submitted). Most of them; however, not all, contain both *ushA*-like and *cpdB*-like genes. According to data in line 44 of Table S6, five *K. pneumoniae* genomes do not contain an *ushA*-like gene, and five do not contain a *cpdB*-like gene. To find out whether they are the same or not, TBlastN results obtained with probes P07024 (UshA protein) and P08331 (CpdB protein) were downloaded and compared in Excel. This comparison indicated that the 1962 hits found with each probe were the same; therefore, it seems that there are five double-negative *K. pneumoniae* genomes, i.e., without both *ushA*-like and *cpdB*-like genes. So, the distribution of these genes in *K. pneumoniae* was near but not fully homogeneous, with a full correlation between them.

In the case of *V. cholerae*, 221 genomes were available in the database, of which 112 gave *ushA*-like and *cpdB*-like hits (line 106 of Table S6). By downloading the TBlastN results obtained for this species with probes P07024 (UshA protein) and P08331 (CpdB protein), it was confirmed that the 112 genomes found by the two probes were the same. Therefore, there are no genomes containing only one of the gene types. All the *V. cholerae* genomes are either double positive or double negative for these genes. Such as in the case of *Salmonella* Typhimurium (see above), the distribution of the genes is clearly not homogeneous; however, with full correlation between both kinds.

#### 4.3.3. About the Variety of Distributions of *ushA*-like and *cpdB*-like Genes in Species of *Streptococcus*

The genus *Streptococcus* is interesting because different species showed different typologies concerning the distribution of *ushA*-like and *cpdB*-like genes (see Table 8 and details in lines 95–104 of Table S6). This includes: complete double positive (*S. sanguinis* and *S. termophilus*, although probes giving complete *ushA* positives were different); complete double negative (*S. mitis* and *S. pneumoniae*); complete positive for *ushA* and negative for *cpdB* (*S. mutans* and *S. pyogenes*, although the positives were obtained with different probes); complete positive for *ushA* and partial for *cpdB* (*S. parasuis*); partial positive for *ushA* and complete positive for *cpdB* (*S. agalactiae*); near complete but not fully positive for both genes (*S. suis*); near complete but not fully positive for *ushA* and partial for *cpdB* (*S. dysgalactiae*). Within *Streptococcus* species, there is both an irregular distribution and an irregular correlation between *ushA*-like and *cpdB*-like genes.

### 4.4. Repercussion of the Results

In the earlier study of the genomic distribution of *cpdB*-like genes [32], the possible repercussions of the different kinds of distribution found (widespread, partial, negative) were analyzed, taking into account the role of CpdB-like proteins in the virulence of pathogens, a feature that is shown both by CpdB-like and UshA-like proteins [21,22,23,24,25,26,27]. Therefore, the same analysis can be applied to the results of the current manuscript. This is summarized in three conclusions (adapted from [32]).

Species that do not contain *ushA*-like and/or *cpdB*-like genes cannot explode the UshA-like or CpdB-like protein-dependent strategies that facilitate innate immunity escape.

Species in which *ushA*-like and/or *cpdB*-like genes are widespread constitute a field to explore the possible role of genes in virulence by creating gene mutants and studying the enzyme activity and specificity of the proteins.

In species with a partial distribution of *ushA*-like and/or *cpdB*-like genes, their presence or absence could modulate the virulence of pathogen strains or isolates.

### 4.5. Strength and Limitations of this Study

The major strength of this study is that it constitutes an extensive catalog of the genomic distribution in *Bacteria* of two genes with related enzymatic function (but different specificities), structure, and role in virulence. Moreover, interesting is that the TBlastN results are analyzed in terms of alignment scores, which are a constant independent of database size.

On the other hand, the following limitations should be considered:

First, the classification of bacterial taxa is eventually subject to alterations, and, in fact, it has been so since the publication of our earlier study [32].

Second, the results obtained for each taxon are not necessarily stable over time. New bacterial genomes are being sequenced and added to the NCBI Complete Genomes Database or, eventually, retired. In some cases, this was observed to occur to a minor extent in the course of data collection. This can affect the results in a significant way for those taxa with few genomes deposited in the database.

Third, to interpret the results of TBlastN searches in terms of the presence of *ushA*-like and *cpdB*-like genes, a minimum alignment score of 151 and a minimum query coverage of 71% were established. This reduces the number of false positives but, in turn, can disregard true but distant homologs. This may have occurred, for instance, with the results of probe WP_011837008, as with some frequency it gave significant scores but with coverages somewhat below 71%.

Fourth, TBlastN hits, even with high scores, reveal the presence of the corresponding genes but do not warrant that they are expressed or that the proteins encoded are enzymatically active. In fact, for instance, silent alleles of *ushA* have been reported in *S. enterica* and *E. coli* [42,43,44,45].

Finally, in such a large collection of data, mistakes are expected. Therefore, in the case of special interest in any concrete result, the readers should be wise to check it by running themselves the relevant TBlastN searches.

## 5. General Summary of Conclusions and Outcomes Worth of Further Investigation

1. An extensive catalog of the distribution of *ushA*-like and *cpdB*-like genes in the genomes of bacterial taxa was constructed by TBlastN analyses of genomes, from phylum to species, run between 30 June and 15 July 2023, with seven different probes, five for *ushA*-like and two for *cpdB*-like genes. In total, the genomes of 43 phyla, 76 classes, 152 orders, 403 families, 510 genera, and 107 species were analyzed. This encompasses every bacterial taxon, since a taxon (class, order, family, or genus) was omitted from analysis only when the genomic analysis of the immediately higher taxonomical level was deemed complete (100% genomes positive and/or negative for both gene kinds).
2. The genomic distributions of both gene kinds are not homogeneous, as while significant homologs occur in many taxa, in many others they do not. For instance, considering only taxa declared complete, 359 contained one or both *ushA*-like and *cpdB*-like genes (Table 10, Table 12, and Table 13), and 231 contained none of them (Table 11).
3. The correlation between both gene kinds is partial, as among complete taxa there were 416 taxa in which both occurred or both were absent (Table 10 and Table 11), while 128 taxa contained only *ushA*-like genes (Table 12), and 46 taxa contained only *cpdB*-like genes (Table 13).
4. One of the probes used for *ushA*-like genes (accession number O32133) revealed the highly frequent occurrence in class *Bacilli* genomes (358/425) of homologs of an uncharacterized *B. subtilis* metallophosphoesterase named YUND_BACSU. This protein is widespread in *B. subtilis* genomes (331/346) and has good homologs in 100% of the genomes of *B. anthracis* and *B. cereus*. These uncharacterized proteins are therefore interesting candidates for cloning, expression, and enzyme characterization. Eventually, they could also be tested for effects on virulence.
5. The five complete genomes available for avian pathogenic *E. coli* (APEC; Table 17) contain high-score hits of *ushA* (probe accession number P070724) and *cpdB* genes (probe accession number P08331). The *cpdB* gene of APEC has been shown previously to be provirulent [30], while the effect of the *ushA* gene has not been investigated. Our data indicate that this is a possibility worth investigating by creating the *ushA* mutant and the double *ushA* and *cpdB* mutants of APEC.
6. The five complete genomes available for *Salmonella* Pullorum contain high-score hits for *ushA* (probe accession number P070724) and *cpdB* genes (probe accession number P08331) (Table S6, line 78). For this *Salmonella* serovar, the *cpdB* gene has been shown previously to be provirulent [31], while the effect of the *ushA* gene has not been investigated. Our data indicate that this is an interesting possibility to explore by creating the single *ushA* mutant and the double *cpdB* and *ushA* mutants of *S.* Pullorum.
7. The different species of the genus *Streptococcus* offer a variety of situations concerning the genomic distribution of *ushA*-like and *cpdB*-like genes (see Section 4.3.3). On the other hand, *ushA*-like genes of *S. sanguinis*, *S. agalactiae*, *S. pyogenes,* and *S. suis*, and *cpdB*-like genes of *S. agalactiae* and *S. suis*, individually considered, have been shown to be pro-virulent for the producing pathogens. However, the following cases remain to be studied: (i) the combined effect of *ushA*-like and *cpdB*-like genes on the virulence of *S. agalactiae* and *S. suis*; (ii) the possible effect of *cpdB*-like genes in the virulence of all the *Streptococcus* species that contain such genes but so far have not been studied in this concern; (iii) the possible effect of *ushA*-like genes in the virulence of all the *Streptococcus* species that contain such genes but so far have not been studied in this concern.

## Figures and Tables

**Table 2:** TBlastN analysis of superkingdom *Bacteria* (taxid:2) with hits recorded among 4185 type-material genomes.

Type of Probe	Probe ^1^	Hits	Score Max.	Score Min.
UshA-like	P07024	630	1099	151
	P44569	456	1188	151
	WP_000726911	468	904	151
	WP_011837008	110	1297	151
	O32133	361	947	171
CpdB-like	P08331	1082	1301	151
	AYV64543	722	1579	153

^1^ The probes are shown in the same background color as Figure 1 and Table 1 to facilitate cross-referencing.

**Table 3:** Presence (+) or absence (−) of *ushA*-like (U) and *cpdB*-like (C) genes in *Bacteria* phyla. The (+) background indicates: green, presence in ≤50% of the genomes analyzed; orange, presence in >50% but <100% of the genomes; red, presence in 100% of the genomes analyzed. Full data can be found in Table S1.

Phylum ^1^	Taxid	U	C
*Abditibacteriota*	2109258	−	−
*Acidobacteriota*	57723	−	−
*Actinomycetota*	201174	+	+
*Aquificota*	200783	−	−
*Armatimonadota*	67819	+	+
*Atribacterota*	67818	+	−
*Bacillota*	1239	+	+
*Bacteroidota*	976	+	+
*Balneolota*	1936987	−	+
*Bdellovibrionota*	3018035	−	−
*Caldisericota*	67814	+	−
*Calditrichota*	1930617	−	+
*Campylobacterota*	29547	+	+
*Chlamydiota*	204428	−	−
*Chlorobiota*	1090	−	−
*Chloroflexota*	200795	+	−
*Chrysiogenota*	200938	*−*	*−*
*Coprothermobacterota*	2138240	−	+
*Cyanobacteriota*	1117	+	*−*
*Deferribacterota*	200930	+	*−*
*Delta/epsilon subdivisions*	68525	+	*−*
*Deinococcota*	1297	+	+
*Dictyoglomota*	68297	+	−
*Elusimicrobiota*	74152	−	−
*Fibrobacterota*	65842	+	−
*Fusobacteriota*	32066	+	+
*Gemmatimonadota*	142182	−	+
*Ignavibacteriota*	1134404	−	+
*Kiritimatiellota*	134625	−	−
*Lentisphaerota*	256845	−	−
*Mycoplasmatota*	544448	+	+
*Myxococcota*	2818505	+	+
*Nitrospinota*	1293497	−	−
*Nitrospirota*	40117	+	−
*Planctomycetota*	203682	−	−
*Pseudomonadota*	1224	+	+
*Rhodothermota*	1853220	−	−
*Spirochaetota*	203691	+	+
*Synergistota*	508458	+	−
*Thermodesulfobacteriota*	200940	+	−
*Thermomicrobiota*	3027942	−	−
*Thermotogota*	200918	+	+
*Verrucomicrobiota*	74201	−	+

^1^ Phyla highlighted in red are not complete according to Section 3.2.1 and are divided into classes in Table 4 and Table S2.

**Table 4:** Presence (+) or absence (−) of *ushA*-like (U) and *cpdB*-like (C) genes in selected bacterial classes. The (+) background indicates: green, presence in ≤50% of the genomes analyzed; orange, presence in >50% but <100% of the genomes; red, presence in all the genomes. Full data are in Table S2.

Phylum	Class ^1^	Taxid	U	C
*Actinomycetota*	*Acidimicrobiia*	84992	+	−
	*Actinomycetes*	1760	+	+
	*Coriobacteriia*	84998	−	−
	*Nitriliruptoria*	908620	+	+
	*Rubrobacteria*	84995	+	−
	*Thermoleophilia*	1497346	+	−
*Armatimonadota*	*Armatimonadia*	1042312	−	−
	*Chthonomonadetes*	1077257	+	−
	*Fimbriimonadia *	1663419	+	−
*Bacillota*	*Bacilli*	91061	+	+
	*Clostridia *	186801	+	+
	*Erysipelotrichia*	526524	+	+
	*Limnochordia*	1676648	+	+
	*Negativicutes *	909932	+	+
	*Tissierellia *	1737404	+	−
*Bacteroidota*	*Bacteroidia*	200643	+	+
	*Chitinophagia*	1853228	−	−
	*Cytophagia*	768503	−	−
	*Flavobacteriia*	117743	−	−
	*Saprospiria*	1937959	−	−
	*Sphingobacteriia*	117747	−	−
*Campylobacterota*	*Desulfurellia*	3031853	−	−
	*Epsilonproteobacteria*	3031852	+	*+*
*Chloroflexota*	*Anaerolineae*	292625	−	*−*
	Ardenticatenia *	1382928	+	*−*
	*Caldilineae*	475962	−	*−*
	*Chloroflexia*	32061	+	*−*
	*Dehalococcoidia*	301297	+	*−*
	*Ktedonobacteria*	388447	−	*−*
	*Tepidiformia*	2682225	−	*−*
	*Thermoflexia*	1495646	+	*−*
*Cyanobacteriota*	*Cyanophyceae*	3028117	+	*−*
*Deferribacterota*	*Deferribacteres*	68337	+	*−*
*Delta/epsilon subdivisions*	*Deltaproteobacteria*	28221	+	*−*
*Deinococcota*	*Deinococci*	188787	+	+
*Dyctioglomota*	*Dictyoglomia*	203486	+	−
*Fibrobacterota*	*Fibrobacteria*	204430	−	−
*Fusobacteriota*	*Fusobacteriia *	203490	+	+
*Gemmatimonadota*	*Gemmatimonadetes*	219685	−	+
	*Longimicrobiia*	1804991	−	−
*Ignavibacteriota*	*Ignavibacteria*	795747	−	+
*Mycoplasmatota*	*Mollicutes*	31969	+	−
	*Mycoplasmoidales*	2790996	−	−
*Myxococcota*	*Myxococcia*	32015	+	+
	*Polyangia*	3031711	+	+
*Nitrospirota*	*Nitrospiria*	203693	+	−
	*Thermodesulfovibrionia*	2811502	−	−
*Pseudomonadota*	*Acidithiobacillia*	1807140	−	−
	*Alphaproteobacteria*	28211	+	+
	*Betaproteobacteria*	28216	+	+
	*Gammaproteobacteria*	1236	+	+
	*Hydrogenophilalia*	2008785	−	−
	*Zetaproteobacteria*	580370	−	−
*Spirochaetota*	*Spirochaetia*	203692	+	+
*Synergistota*	*Synergistia*	649775	+	−
*Thermodesulfobacteriota*	*Desulfarculia*	3031646	−	−
	*Desulfobaccia*	3031647	−	−
	*Desulfobacteria*	3024418	+	−
	*Desulfobulbia*	3031451	+	−
	*Desulfomonilia*	3031650	+	−
	*Desulfovibrionia*	3031449	+	−
	*Desulfuromonadia*	3031651	+	−
	*Syntrophia*	3031648	+	−
	*Syntrophobacteria *	3024408	+	−
	*Thermodesulfobacteria*	67799	+	−
*Thermotogota*	*Thermotogae*	188708	+	+
*Verrucomicrobiota*	*Methylacidiphilae*	1955630	−	−
	*Opitutae*	414999	+	+
	*Spartobacteria*	134549	−	−
	*Verrucomicrobiae*	203494	−	−

^1^ Classes highlighted in red are not complete according to Section 3.2.1 and are divided into orders in Table 5 and Table S3, except for one *. * The class Ardenticatenia does not appear in Table 5 because the single hit obtained in the TBlastN analysis corresponds to a “candidatus” order.

**Table 5:** Presence (+) or absence (−) of *ushA*-like (U) and *cpdB*-like (C) genes in selected bacterial orders. The (+) background indicates: green, presence in ≤50% of the genomes analyzed; orange, presence in >50% but <100% of the genomes; red, presence in all the genomes. Full data can be found in Table S3.

Class	Order ^1^	Taxid	U	C
*Acidimicrobiia*	*Acidimicrobiales*	84993	+	−
*Actinomycetes*	*Acidothermales*	1643683	−	−
	*Actinomycetales*	2037	−	+
	*Actinopolysporales*	622450	−	−
	*Bifidobacteriales*	85004	−	−
	*Catenulisporales*	414714	−	−
	*Frankiales*	85013	−	−
	*Geodermatophilales*	1643682	+	−
	*Glycomycetales*	85014	+	−
	*Jatrophihabitantales*	2805415	+	+
	*Kineosporiales*	622452	−	+
	*Kitasatosporales*	85011	+	+
	*Micrococcales*	85006	+	+
	*Micromonosporales*	85008	+	+
	*Mycobacteriales*	85007	+	−
	*Nakamurellales*	1643684	−	−
	*Propionibacteriales*	85009	+	+
	*Pseudonocardiales*	85010	+	+
	*Sporichthyales*	2495578	−	+
	*Streptosporangiales*	85012	+	+
*Nitriliruptoria*	*Egibacterales*	1747768	−	−
	*Egicoccales*	1755823	+	+
	*Euzebyales*	908621	−	−
*Rubrobacteria*	*Rubrobacterales*	84996	+	−
*Thermoleophilia*	*Miltoncostaeales*	2843198	+	−
	*Solirubrobacterales*	588673	−	−
	*Thermoleophilales*	588674	−	−
*Fimbriimonaadia*	*Fimbriimonadales*	1663425	+	−
*Bacilli*	*Bacillales*	1385	+	+
	*Lactobacillales*	186826	+	+
*Clostridia*	*Eubacteriales*	186802	+	+
	*Halanaerobiales*	53433	+	+
	*Koleobacterales*	2786987	−	−
	*Moorellales*	3039167	−	−
	*Natranaerobiales*	485256	−	−
	*Thermoanaerobacterales*	68295	+	+
	*Thermosediminibacterales*	2770089	+	+
*Erysipelotrichia*	*Erysipelotrichales*	526525	+	+
*Negativicutes*	*Acidaminococcales*	1843488	−	−
	*Selenomonadales*	909929	+	+
	*Veillonellales*	1843489	+	−
*Tissierella*	*Tissierellales*	1737405	+	−
*Bacteroidia*	*Bacteroidales*	171549	+	+
	*Marinilabiliales*	1970189	−	−
*Epsilonproteobacteria*	*Campylobacterales*	213849	+	+
	*Nautiliales*	235899	+	−
*Dehalococcoidia*	*Dehalococcoidales*	1202465	−	−
	*Dehalogenimonas*	670486	−	−
*Cyanophyceae*	*Chroococcidiopsidales*	1890505	−	−
	*Gloeobacterales*	307595	−	−
	*Gloeomargaritales*	1955042	+	−
	*Nostocales*	1161	+	−
	*Chroococcales*	1118	−	−
	*Oscillatoriales*	1150	+	−
	*Pleurocapsales*	52604	−	−
	*Pseudanabaenales*	2881377	−	−
	*Synechococcales*	1890424	−	−
	*Thermostichales*	2881383	−	−
*Deferribacteres*	*Deferribacterales*	191393	+	−
*Deltaproteobacteria*	*Bradymonadales*	1779134	+	−
*Deinococci*	*Deinococcales*	118964	+	+
	*Thermales*	68933	+	−
	*Trueperales*	2762275	−	−
*Fusobacteriia*	*Fusobacteriales*	203491	+	+
*Gemmatimonadetes*	*Gemmatimonadales*	219686	−	+
*Ignavibacteria*	*Ignavibacteriales*	795748	−	+
*Mollicutes*	*Acholeplasmatales*	186329	−	−
	*Entomoplasmatales*	186328	−	−
	*Mycoplasmatales*	2085	*+*	*−*
*Myxococcia*	*Myxococcales*	29	+	+
*Polyangia*	*Haliangiales*	3031714	−	+
	*Nannocystales*	3031713	+	+
	*Polyangiales*	3031712	+	+
*Nitrospiria*	*Nitrospirales*	189778	+	−
*Alphaproteobacteria*	*Caulobacterales*	204458	−	−
	*Emcibacterales*	2066490	−	−
	*Holosporales*	1921002	−	−
	*Hyphomicrobiales*	356	+	+
	*Hyphomonadales*	2800060	+	−
	*Kordiimonadales*	362534	−	−
	*Magnetococcales*	1191478	−	−
	*Maricaulales*	2800059	−	−
	*Minwuiales*	2493627	−	−
	*Parvularculales*	255473	−	−
	*Rhodobacterales*	204455	+	+
	*Rhodospirillales*	204441	+	+
	*Rickettsiales*	766	−	−
	*Sneathiellales*	510684	−	−
	*Sphingomonadales*	204457	+	−
*Betaproteobacteria*	*Burkholderiales*	80840	+	+
	*Ferrovales*	1442155	−	−
	*Neisseriales*	206351	+	+
	*Nitrosomonadales*	32003	+	−
	*Rhodocyclales*	206389	+	+
*Gammaproteobacteria*	*Acidiferrobacterales*	1692040	−	−
	*Aeromonadales*	135624	+	+
	*Alteromonadales*	135622	+	+
	*Cardiobacteriales*	135615	−	+
	*Cellvibrionales*	1706369	+	+
	*Chromatiales*	135613	+	+
	*Enterobacterales*	91347	+	+
	*Immundisolibacterales*	1934945	+	−
	*Kangiellales*	2887327	−	−
	*Legionellales*	118969	+	−
	*Methylococcales*	135618	+	−
	*Moraxellales*	2887326	+	+
	*Nevskiales*	1775403	+	−
	*Oceanospirillales*	135619	+	+
	*Orbales*	1240482	+	+
	*Pasteurellales*	135625	*+*	+
	*Pseudomonadales*	72274	+	−
	*Thiotrichales*	72273	+	+
	*Vibrionales*	135623	+	+
	*Xanthomonadales*	135614	−	+
*Spirochaetia*	*Brachyspirales*	1643686	+	−
	*Brevinematales*	1643687	−	−
	*Leptospirales*	1643688	−	−
	*Spirochaetales*	136	+	+
*Synergistia*	*Synergistales*	649775	+	−
*Desulfobacteria*	*Desulfobacterales*	213118	+	−
*Desulfobulbia*	*Desulfobulbales*	3024411	+	−
*Desulfovibrionia*	*Desulfovibrionales*	213115	+	−
*Desulfuromonadia*	*Desulfuromonadales*	69541	+	−
	*Geobacterales*	3031668	+	−
*Syntrophobacteria*	*Syntrophobacterales*	213462	+	−
*Thermodesulfobacteria*	*Thermodesulfobacteriales*	188710	+	−
*Thermotogae*	*Kosmotogales*	1643946	+	+
	*Mesoaciditogales*	1769716	+	+
	*Petrotogales*	1643947	+	+
	*Thermotogales*	2419	+	+
*Opitutae*	*Opitutales*	415000	+	+
	*Puniceicoccales*	415001	−	−

^1^ Orders highlighted in red are not complete according to Section 3.2.1 and are divided into families in Table 6 and Table S4.

**Table 6:** Presence (+) or absence (−) of *ushA*-like (U) and *cpdB*-like (C) genes in selected bacterial families. The (+) background indicates: green, presence in ≤50% of the genomes analyzed; orange, presence in >50% but <100% of the genomes; red, presence in all the genomes. Full data can be found in Table S4.

Order	Family ^1^	Taxid	U	C
*Acidimicrobiales*	*Acidimicrobiaceae*	84994	−	−
	*Iamiaceae*	633392	−	−
	*Ilumatobacteraceae*	2448023	+	−
*Actinomycetales*	*Actinomycetaceae*	2049	−	+
*Geodermathophilales*	*Geodermatophilaceae*	85030	+	−
*Jatrophihabitantales*	*Jatrophihabitantaceae*	2805416	+	+
*Kineosporiales*	*Kineosporiaceae*	83778	−	+
*Kitasatosporales*	*Streptomycetaceae*	2062	+	+
*Micrococcales*	*Beutenbergiaceae*	125316	−	−
	*Bogoriellaceae*	145358	+	+
	*Brevibacteriaceae*	85019	+	+
	*Cellulomonadaceae*	85016	−	−
	*Demequinaceae*	1042322	−	−
	*Dermabacteraceae*	85020	−	+
	*Dermacoccaceae*	145357	+	+
	*Dermatophilaceae*	85018	+	−
	*Intrasporangiaceae*	85021	+	+
	*Jonesiaceae*	85022	−	−
	*Kytococcaceae*	2805426	+	−
	*Microbacteriaceae*	85023	+	+
	*Micrococcaceae*	1268	+	+
	*Ornithinimicrobiaceae*	2805590	+	−
	*Promicromonosporaceae*	85017	+	−
	*Ruaniaceae*	1331736	+	+
	*Sanguibacteraceae*	145360	+	−
	*Tropherymataceae*	2805591	−	−
*Micromonosporales*	*Micromonosporaceae*	28056	+	+
*Mycobacteriales*	*Corynebacteriaceae*	1653	+	−
	*Dietziaceae*	85029	−	−
	*Gordoniaceae*	85026	−	−
	*Hoyosellaceae*	3040680	−	−
	*Lawsonellaceae*	2805586	−	−
	*Mycobacteriaceae*	1762	−	−
	*Nocardiaceae*	85025	+	−
	*Segniliparaceae*	316606	−	−
	*Tsukamurellaceae*	85028	+	−
*Propionibacteriales*	*Kribbellaceae*	2726069	+	+
	*Nocardioidaceae*	85015	+	+
	*Propionibacteriaceae*	31957	−	+
*Pseudonocardiales*	*Pseudonocardiaceae*	2070	+	+
*Streptosporangiales*	*Nocardiopsaceae*	83676	−	−
	*Streptosporangiaceae*	2004	+	+
	*Thermomonosporaceae*	2012	+	+
*Rubrobacterales*	*Baekduiaceae*	2600303	+	−
	*Rubrobacteraceae*	84997	+	−
*Miltoncostaeales*	*Miltoncostaeaceae*	2843199	+	−
*Fimbriimonadales*	*Fimbriimonadaceae*	1663426	−	−
*Bacillales*	*Alicyclobacillaceae*	186823	+	+
	*Bacillaceae*	186817	+	+
	*Listeriaceae*	186820	+	−
	*Paenibacillaceae*	186822	+	+
	*Planococcaceae*	186818	+	+
	*Sporolactobacillaceae*	186821	+	+
	*Staphylococcaceae*	90964	+	+
	*Thermoactinomycetaceae*	186824	+	+
*Lactobacillales*	*Aerococcaceae*	186827	+	+
	*Carnobacteriaceae*	186828	+	+
	*Enterococcaceae*	81852	+	+
	*Lactobacillaceae*	33958	+	+
	*Streptococcaceae*	1300	+	+
*Eubacteriales*	*Aristaeellaceae*	3046368	−	−
	*Cellulosilyticaceae*	3018741	−	−
	*Christensenellaceae*	990719	−	−
	*Clostridiaceae*	31979	+	+
	*Desulfallaceae*	2867375	−	−
	*Desulfitobacteriaceae*	2937909	−	+
	*Desulfotomaculaceae*	2937910	−	−
	*Eubacteriaceae*	186806	+	−
	*Heliobacteriaceae*	31984	−	−
	*Lachnospiraceae*	186803	+	+
	*Maliibacteriaceae*	3047432	−	−
	*Oscillospiraceae*	216572	+	+
	*Peptococcaceae*	186807	−	−
	*Peptostreptococcaceae*	186804	+	−
	*Proteinivoraceae*	1491775	−	−
	*Symbiobacteriaceae*	543349	−	−
	*Syntrophomonadaceae*	68298	−	−
	*Thermincolaceae*	2937911	−	−
	*Vallitaleaceae*	2603322	+	+
*Halanaerobiales*	*Halanaerobiaceae*	972	+	+
	*Halarsenatibacteraceae*	3046411	−	−
	*Halobacteroidaceae*	53434	+	+
	*Halothermotrichaceae*	3046412	−	−
*Thermoanaerobacterales*	*Thermoanaerobacteraceae*	186814	+	+
	*Thermodesulfobiaceae*	227387	−	−
*Thermosediminibacterales*	*Tepidanaerobacteraceae*	2770092	−	−
	*Thermosediminibacteraceae*	2770093	+	+
*Erysipelotrichales*	*Coprobacillaceae*	2810280	−	−
	*Erysipelotrichaceae*	128827	+	+
	*Turicibacteraceae*	2810281	+	−
*Selenomonadales*	*Selenomonadaceae*	1843491	+	+
	*Sporomusaceae*	1843490	+	+
*Veillonellales*	*Veillonellaceae*	31977	+	−
*Tissierellales*	*Acidilutibacteraceae*	2992717	−	−
	*Gottschalkiaceae*	2042895	+	−
	*Peptoniphilaceae*	1570339	−	−
	*Tepidimicrobiaceae*	2992719	+	−
	*Thermohalobacteraceae*	2848916	+	−
	*Tissierellaceae*	1737406	+	+
*Bacteroidales*	*Bacteroidaceae*	815	−	+
	*Barnesiellaceae*	2005519	+	+
	*Dysgonomonadaceae*	2005520	−	−
	*Muribaculaceae*	2005473	−	+
	*Odoribacteraceae*	1853231	+	−
	*Paludibacteraceae*	2005523	−	−
	*Porphyromonadaceae*	171551	−	+
	*Prevotellaceae*	171552	−	+
	*Rikenellaceae*	171550	−	−
	*Salinivirgaceae*	1970190	−	−
	*Tannerellaceae*	2005525	−	+
	*Tenuifilaceae*	2760872	−	−
*Campylobacterales*	*Arcobacteraceae*	2808963	+	−
	*Campylobacteraceae*	72294	+	−
	*Helicobacteraceae*	72293	−	+
	*Hydrogenimonadaceae*	292630	−	−
	*Sulfurimonadaceae*	2771471	−	−
	*Sulfurospirillaceae*	2932623	−	−
	*Sulfurovaceae*	2771472	−	−
*Nautiliales*	*Nautiliaceae*	224467	+	−
	*Nitratiruptoraceae*	2795691	−	−
*Nostocales*	*Aphanizomenonaceae*	1892259	−	−
	*Calotrichaceae*	2661849	+	−
	*Hapalosiphonaceae*	1892263	−	−
	*Nostocaceae*	1162	−	−
	*Rivulariaceae*	1185	−	−
	*Tolypothrichaceae*	119859	−	−
*Oscillatoriales*	*Coleofasciculaceae*	1892251	−	−
	*Gomontiellaceae*	1892255	−	−
	*Microcoleaceae*	1892252	+	−
	*Oscillatoriaceae*	1892254	−	−
*Deferribacterales*	*Calditerrivibrionaceae*	2945021	−	−
	*Deferribacteraceae*	191394	−	−
	*Flexistipitaceae*	2945022	−	−
	*Geovibrionaceae*	2945019	+	−
	*Mucispirillaceae*	2945020	−	−
*Deinococcales*	*Deinococcaceae*	183710	+	+
*Thermales*	*Thermaceae*	188786	+	*−*
*Fusobacteriales*	*Fusobacteriaceae*	203492	+	*−*
	*Leptotrichiaceae*	1129771	+	*+*
*Gemmatimonadales*	*Gemmatimonadaceae*	219687	−	+
*Ignavibacteriales*	*Ignavibacteriaceae*	795749	−	+
	*Melioribacteraceae*	1334117	−	−
*Mycoplasmatales*	*Mycoplasmataceae*	2092	+	−
*Myxococcales*	*Anaeromyxobacteraceae*	1524215	−	−
	*Archangiaceae*	39	+	+
	*Myxococcaceae*	31	+	+
	*Vulgatibacteraceae*	1524213	−	+
*Polyangiales*	*Labilitrichaceae*	1524216	−	−
	*Polyangiaceae*	49	+	+
	*Sandaracinaceae*	1055686	−	−
*Nitrospirales*	*Nitrospiraceae*	189779	+	−
*Hyphomicrobiales*	*Amorphaceae*	2685818	−	−
	*Aurantimonadaceae*	255475	+	+
	*Bartonellaceae*	772	−	−
	*Beijerinckiaceae*	45404	−	−
	*Blastochloridaceae*	2831090	−	−
	*Boseaceae*	2831100	−	+
	*Breoghaniaceae*	2831104	+	+
	*Brucellaceae*	118882	+	−
	*Chelatococcaceae*	2036754	−	−
	*Devosiaceae*	2831106	+	+
	*Hyphomicrobiaceae*	45401	−	−
	*Kaistiaceae*	2831111	−	−
	*Lichenihabitantaceae*	2723775	−	−
	*Methylobacteriaceae*	119045	+	+
	*Methylocystaceae*	31993	−	−
	*Nitrobacteraceae*	41294	+	−
	*Parvibaculaceae*	2813035	−	−
	*Phreatobacteraceae*	2843305	−	−
	*Phyllobacteriaceae*	69277	+	+
	*Pleomorphomonadaceae*	2843308	+	−
	*Rhizobiaceae*	82115	+	+
	*Stappiaceae*	2821832	+	+
	*Xanthobacteraceae*	335928	−	−
*Hyphomonadales*	*Hyphomonadaceae*	69657	+	−
*Rhodobacterales*	*Paracoccaceae*	31989	+	+
	*Roseobacteraceae*	2854170	+	+
*Rhodospirillales*	*Acetobacteraceae*	433	+	+
	*Azospirillaceae*	2829815	+	+
	*Elioraeaceae*	2690195	+	−
	*Geminicoccaceae*	2066434	−	−
	*Kiloniellaceae*	597359	+	+
	*Rhodospirillaceae*	41295	−	−
	*Stellaceae*	2844601	−	−
	*Terasakiellaceae*	2813951	−	−
	*Thalassobaculaceae*	2844864	−	−
	*Thalassospiraceae*	2844866	+	+
*Sphingomonadales*	*Erythrobacteraceae*	335929	+	−
	*Sphingomonadaceae*	41297	+	−
	*Sphingosinicellaceae*	2820280	+	−
	*Zymomonadaceae*	2844881	−	−
*Burkholderiales*	*Alcaligenaceae*	506	+	+
	*Burkholderiaceae*	119060	+	+
	*Comamonadaceae*	80864	+	+
	*Oxalobacteraceae*	75682	+	+
	*Sphaerotilaceae*	2975441	+	+
	*Sutterellaceae*	995019	+	−
*Neisseriales*	*Chromobacteriaceae*	1499392	+	+
	*Neisseriaceae*	481	+	+
*Nitrosomonadales*	*Gallionellaceae*	90627	−	−
	*Methylophilaceae*	32011	−	−
	*Nitrosomonadaceae*	206379	−	−
	*Sterolibacteriaceae*	2008793	−	−
	*Sulfuricellaceae*	2772226	−	−
	*Thiobacillaceae*	2008790	+	−
	*Usitatibacteraceae*	2803844	+	−
*Rhodocyclales*	*Azonexaceae*	2008795	+	−
	*Fluviibacteraceae*	2808923	−	−
	*Rhodocyclaceae*	75787	+	+
	*Zoogloeaceae*	2008794	+	−
*Aeromonadales*	*Aeromonadaceae*	84642	+	+
	*Succinivibrionaceae*	83763	−	−
*Alteromonadales*	*Alteromonadaceae*	72275	+	+
	*Colwelliaceae*	267889	+	−
	*Ferrimonadaceae*	267892	+	+
	*Idiomarinaceae*	267893	+	+
	*Moritellaceae*	267891	+	+
	*Pseudoalteromonadaceae*	267888	+	+
	*Psychromonadaceae*	267894	+	+
	*Shewanellaceae*	267890	+	+
*Cardiobacteriales*	*Cardiobacteriaceae*	868	−	−
	*Ignatzschineriaceae*	3018589	−	+
*Cellvibrionales*	*Cellvibrionaceae*	1706371	+	+
	*Halieaceae*	1706372	−	−
	*Microbulbiferaceae*	1706373	+	+
	*Porticoccaceae*	1706374	−	−
	*Spongiibacteraceae*	1706375	+	−
*Chromatiales*	*Chromatiaceae*	1046	+	−
	*Ectothiorhodospiraceae*	72276	+	−
	*Granulosicoccaceae*	449719	+	+
	*Halothiobacillaceae*	255526	−	−
	*Thioalkalibacteraceae*	2035710	−	−
	*Thioalkalispiraceae*	1096778	−	*−*
	*Wenzhouxiangellaceae*	1676141	−	*−*
	*Woeseiaceae*	1738654	−	*−*
*Enterobacterales*	*Bruguierivoracaceae*	2812006	−	*−*
	*Budviciaceae*	1903416	−	*+*
	*Enterobacteriaceae*	543	+	+
	*Erwiniaceae*	1903409	+	+
	*Hafniaceae*	1903412	+	+
	*Morganellaceae*	1903414	+	+
	*Pectobacteriaceae*	1903410	+	+
	*Yersiniaceae*	1903411	+	+
*Legionellales*	*Coxiellaceae*	118968	−	−
	*Legionellaceae*	444	+	−
*Methylococcales*	*Methylococcaceae*	403	+	−
	*Methylothermaceae*	1486721	−	−
*Moraxellales*	*Moraxellaceae*	468	+	+
*Nevskiales*	*Nevskiaceae*	568386	+	–
	*Steroidobacteraceae*	2689614	–	–
*Oceanospirillales*	*Alcanivoracaceae*	224372	+	+
	*Endozoicomonadaceae*	2066474	+	+
	*Hahellaceae*	224379	+	−
	*Halomonadaceae*	28256	+	+
	*Litorivicinaceae*	449732	−	−
	*Oceanospirillaceae*	135620	+	+
	*Oleiphilaceae*	191033	+	+
	*Saccharospirillaceae*	255527	+	+
	*Zooshikellaceae*	2898533	+	−
*Orbales*	*Orbaceae*	1240483	+	+
*Pasteurellales*	*Pasteurellaceae*	712	+	+
*Pseudomonadales*	*Marinobacteraceae*	2887365	−	−
	*Pseudomonadaceae*	135621	+	−
*Thiotrichales*	*Fastidiosibacteraceae*	2056687	−	−
	*Francisellaceae*	34064	−	−
	*Piscirickettsiaceae*	135616	+	−
	*Thiotrichaceae*	135617	+	+
*Vibrionales*	*Vibrionaceae*	641	+	+
*Xanthomonadales*	*Rhodanobacteraceae*	1775411	−	+
	*Xanthomonadaceae*	32033	−	+
*Brachyspirales*	*Brachyspiraceae*	143786	+	−
*Spirochaetales*	*Borreliaceae*	1643685	−	−
	*Breznakiellaceae*	2951104	+	+
	*Sphaerochaetaceae*	2791015	+	+
	*Spirochaetaceae*	137	+	+
	*Treponemataceae*	2845253	+	+
*Synergistales*	*Acetomicrobiaceae*	3029086	−	−
	*Aminithiophilaceae*	3029085	+	−
	*Aminobacteriaceae*	3029087	−	−
	*Dethiosulfovibrionaceae*	3029088	−	−
	*Synergistaceae*	649777	+	−
	*Thermovirgaceae*	3029089	−	−
*Desulfobacterales*	*Desulfatibacillaceae*	3031627	+	−
	*Desulfobacteraceae*	213119	+	−
	*Desulfococcaceae*	2931039	−	−
	*Desulfolunaceae*	3031622	−	−
	*Desulfosarcinaceae*	3031624	+	−
	*Desulfosudaceae*	2904715	−	−
*Desulfobulbales*	*Desulfobulbaceae*	213121	+	−
	*Desulfocapsaceae*	2886822	+	−
*Desulfovibrionales*	*Desulfohalobiaceae*	213117	−	−
	*Desulfomicrobiaceae*	213116	+	−
	*Desulfovibrionaceae*	194924	+	−
*Desulfuromonadales*	*Desulfuromonadaceae*	213421	+	−
	*Geoalkalibacteraceae*	3031665	−	−
	*Syntrophotaleaceae*	2812024	−	−
*Geobacterales*	*Geobacteraceae*	213422	+	−
*Syntrophobacteriales*	*Syntrophobacteraceae*	213465	+	−
*Thermodesulfobacteriales*	*Thermodesulfatatoraceae*	3031464	+	−
	*Thermodesulfobacteriaceae*	188711	−	−
*Petrotogales*	*Petrotogaceae*	1643949	+	+
*Thermotogales*	*Fervidobacteriaceae*	1643950	+	+
	*Thermotogaceae*	188709	+	−
*Opitutales*	*Opitutaceae*	134623	+	+

^1^ Families highlighted in red are not complete according to Section 3.2.1 and are divided into genera in Table 7 and Table S5.

**Table 7:** Presence (+) or absence (−) of *ushA*-like (U) and *cpdB*-like (C) genes in selected bacterial genera. The (+) background indicates: green, presence in ≤50% of the genomes analyzed; orange, presence in >50% but <100% of the genomes; red, presence in all the genomes. Full data can be found in Table S5.

Family	Genus ^1^	Taxid	U	C
*Actinomycetaceae*	*Flaviflexus*	1522056	−	+
*Geodermatophilaceae*	*Blastococcus*	38501	+	−
	*Geodermatophilus*	1860	+	−
	*Modestobacter*	88138	+	−
*Jatrophihabitantaceae*	*Jatrophihabitans*	1434010	+	+
*Kineosporiaceae*	*Kineococcus*	33981	−	+
*Streptomycetaceae*	*Kitasatospora*	2063	+	+
	*Peterkaempfera*	2995704	+	+
	*Streptantibioticus*	2995706	−	+
	*Streptomyces*	1883	+	+
*Bogoriellaceae*	*Georgenia*	154116	+	+
*Brevibacteriaceae*	*Brevibacterium*	1696	+	+
*Dermabacteraceae*	*Dermabacter*	36739	−	+
*Dermacoccaceae*	*Luteipulveratus*	745364	+	+
*Dermatophilaceae*	*Austwickia*	1184606	+	−
	*Dermatophilus*	1862	+	−
*Intrasporangiaceae*	*Arsenicicoccus*	267408	+	−
	*Intrasporangium*	53357	+	+
	*Janibacter*	53457	+	−
	*Phycicoccus*	367298	+	+
	*Tetrasphaera*	99479	−	+
*Microbacteriaceae*	*Clavibacter*	1573	+	−
	*Microbacterium*	33882	+	+
	*Protaetiibacter*	2680004	+	−
	*Rathayibacter*	33886	−	+
*Micrococcaceae*	*Arthrobacter*	1663	+	+
*Ornithinimicrobiaceae*	*Ornithinimicrobium*	125287	+	−
	*Serinicoccus*	265976	+	−
*Promicromonosporaceae*	*Isoptericola*	254250	+	−
*Ruaniaceae*	*Occultella*	2828348	+	+
	*Ruania*	626119	+	−
*Sanguibacteraceae*	*Sanguibacter*	60919	+	−
*Micromonosporaceae*	*Actinocatenispora*	390988	−	+
	*Actinoplanes*	1865	+	+
	*Dactylosporangium*	35753	+	+
	*Micromonospora*	1873	+	+
	*Phytohabitans*	907364	+	−
	*Salinispora*	168694	+	−
*Corynebacteriaceae*	*Corynebacterium*	1716	+	+
*Nocardiaceae*	*Nocardia*	1817	+	−
	*Rhodococcus*	1827	+	−
*Tsukamurellaceae*	*Tsukamurella*	2060	+	−
*Nocardioidaceae*	*Nocardioides*	1839	+	+
*Propionibacteriaceae*	*Cutibacterium*	1912216	−	+
*Pseudonocardiaceae*	*Actinosynnema*	40566	+	−
	*Amycolatopsis*	1813	+	−
	*Kibdelosporangium*	2029	+	−
	*Kutzneria*	43356	+	+
	*Lentzea*	165301	+	+
	*Pseudonocardia*	1847	+	−
	*Saccharopolyspora*	1835	+	−
	*Saccharothrix*	2071	+	+
*Streptosporangiaceae*	*Nonomuraea*	83681	+	+
	*Streptosporangium*	2000	+	+
	*Thermobispora*	147067	+	−
*Thermomonosporaceae*	*Actinomadura*	1988	+	+
*Baekduiaceae*	*Baekduia*	2600304	+	−
*Rubrobacteraceae*	*Rubrobacter*	42255	+	−
*Miltoncostaeaceae*	*Miltoncostaea*	2843200	+	−
*Alicyclobacillaceae*	*Alicyclobacillus*	29330	+	+
	*Effusibacillus*	1502725	+	−
	*Tumebacillus*	432330	+	−
*Bacillaceae*	*Alkalihalobacillus*	2675234	+	+
	*Allobacillus*	1400133	+	+
	*Amphibacillus*	29331	+	−
	*Anaerobacillus*	704093	+	−
	*Anoxybacillus*	150247	+	+
	*Bacillus*	1386	+	+
	*Cytobacillus*	2675230	+	+
	*Evansella*	2837485	+	+
	*Fervidibacillus*	3033930	+	−
	*Geobacillus*	129337	+	+
	*Gracilibacillus*	74385	+	+
	*Halalkalibacter*	2893056	+	−
	*Halobacillus*	45667	+	+
	*Heyndrickxia*	2837504	+	+
	*Lederbergia*	2804231	+	−
	*Lentibacillus*	175304	+	+
	*Lysinibacillus*	400634	+	+
	*Mangrovibacillus*	2920444	+	+
	*Metabacillus*	2675233	+	+
	*Oceanobacillus*	182709	+	+
	*Paenalkalicoccus*	2944627	+	+
	*Parageobacillus*	1906945	+	−
	*Paraliobacillus*	200903	+	+
	*Peribacillus*	2675229	+	+
	*Priestia*	2800373	+	+
	*Psychrobacillus*	1221880	+	+
	*Radiobacillus*	2785518	+	+
	*Salicibibacter*	2685905	+	+
	*Salimicrobium*	351195	+	−
	*Salisediminibacterium*	1434042	+	+
	*Sediminibacillus*	482460	+	+
	*Sutcliffiella*	2837511	+	+
	*Virgibacillus*	84406	+	+
	*Weizmannia*	2817139	+	+
*Paenibacillaceae*	*Aneurinibacillus*	55079	+	−
	*Brevibacillus*	55080	+	+
	*Cohnella*	329857	+	+
	*Paenibacillus*	44249	+	+
	*Saccharibacillus*	456492	+	+
	*Thermobacillus*	76632	+	−
*Planococcaceae*	*Jeotgalibacillus*	157226	+	−
	*Paenisporosarcina*	651660	+	+
	*Planococcus*	1372	+	+
	*Sporosarcina*	1569	+	+
*Sporolactobacillaceae*	*Pullulanibacillus*	475230	+	−
	*Sporolactobacillus*	2077	+	+
*Staphylococcaceae*	*Macrococcus*	69965	+	+
	*Mammaliicoccus*	2803850	+	+
	*Nosocomiicoccus*	489909	+	−
	*Staphylococcus*	1279	+	+
*Thermoactinomycetaceae*	*Kroppenstedtia*	1274351	+	+
	*Polycladomyces*	1348505	+	+
	*Novibacillus*	1677050	+	−
	*Staphylospora*	2689589	+	−
*Lactobacillaceae*	*Acetilactobacillus*	2767874	+	+
	*Amylolactobacillus*	2767876	+	+
	*Apilactobacillus*	2767877	+	+
	*Bombilactobacillus*	2767878	+	+
	*Companilactobacillus*	2767879	+	−
	*Fructilactobacillus*	2767881	+	+
	*Fructobacillus*	559173	+	+
	*Lacticaseibacillus*	2759736	+	+
	*Lactiplantibacillus*	2767842	+	+
	*Lactobacillus*	1578	+	+
	*Latilactobacillus*	2767885	+	−
	*Lentilactobacillus*	2767893	+	+
	*Leuconostoc*	1243	+	+
	*Levilactobacillus*	2767886	+	+
	*Limosilactobacillus*	2742598	+	−
	*Loigolactobacillus*	2767889	+	+
	*Nicoliella*	2978367	+	+
	*Oenococcus*	46254	+	+
	*Paucilactobacillus*	2767890	+	−
	*Pediococcus*	1253	−	+
	*Periweissella*	2930384	+	+
	*Weissella*	46255	+	+
*Streptococcaceae*	*Lactococcus*	1357	+	+
	*Streptococcus*	1301	+	+
*Enterococcaceae*	*Enterococcus*	1350	+	+
	*Tetragenococcus*	51668	+	−
	*Vagococcus*	2737	+	+
*Carnobacteriaceae*	*Carnobacterium*	2747	+	+
	*Granulicatella*	117563	+	+
	*Jeotgalibaca*	1470540	+	−
*Aerococcaceae*	*Aerococcus*	1375	+	+
	*Suicoccus*	2689587	+	−
*Clostridiaceae*	*Alkaliphilus*	114627	+	+
	*Clostridium*	1485	+	+
	*Crassaminicella*	1848399	+	−
	*Geosporobacter*	390805	+	−
	*Hathewaya*	1769729	+	−
	*Paraclostridium*	1849822	+	+
*Desulfitobacteriaceae*	*Desulfosporosinus*	79206	−	+
*Eubacteriaceae*	*Eubacterium*	1730	+	−
*Lachnospiraceae*	*Anaerocolumna*	1843210	+	−
	*Anaeromicropila*	3024823	−	+
	*Anaeropeptidivorans*	2997360	+	−
	*Herbinix*	1663717	−	+
	*Lachnoclostridium*	1506553	−	+
	*Tyzzerella*	1506577	−	+
*Oscillospiraceae*	*Anaerotruncus*	244127	+	+
	*Flavonifractor*	946234	+	+
	*Ruminiclostridium*	1508657	+	−
	*Vescimonas*	2892396	−	+
*Peptostreptococcaceae*	*Acetoanaerobium*	186831	+	−
	*Clostridioides*	1870884	+	−
*Vallitaleaceae*	*Petrocella*	2603323	+	+
	*Vallitalea*	1348611	+	+
*Halanaerobiaceae*	*Halanaerobium*	2330	+	+
	*Halocella*	46466	+	+
	*Iocasia*	2899804	+	+
*Halobacteroidaceae*	*Halobacteroides*	42417	+	+
*Thermoanaerobacteraceae*	*Aceticella*	3051499	+	−
	*Caldanaerobacter*	249529	−	+
	*Carboxydothermus*	129957	+	+
	*Thermoanaerobacter*	1754	+	+
	*Thermoanaerobacterium*	28895	−	+
*Erysipelotrichaceae*	*Erysipelothrix*	1647	+	+
*Turicibacteraceae*	*Turicibacter*	191303	+	−
*Selenomonadaceae*	*Selenomonas*	970	+	+
*Sporomusaceae*	*Methylomusa*	2093783	+	+
	*Pelosinus*	365348	+	−
*Veillonellaceae*	*Megasphaera*	906	+	−
*Tissierellaceae*	*Gudongella*	2692382	+	−
	*Tissierella*	41273	+	+
*Bacteroidaceae*	*Bacteroides*	816	−	+
	*Phocaeicola*	909656	−	+
*Barnesiellaceae*	*Barnesiella*	397864	+	−
	*Coprobacter*	1348911	−	+
*Muribaculaceae*	*Duncaniella*	2518495	−	+
	*Muribaculum*	1918540	−	+
	*Sodaliphilus*	2815786	−	+
*Odoribacteraceae*	*Odoribacter*	283168	+	−
*Porphyromonadaceae*	*Porphyromonas*	836	−	+
*Prevotellaceae*	*Prevotella*	838	−	+
	*Pseudoprevotella*	2884814	−	+
*Tannerellaceae*	*Parabacteroides*	375288	−	+
*Arcobacteraceae*	*Arcobacter*	28196	+	−
	*Malaciobacter*	2321114	+	−
*Campylobacteraceae*	*Campylobacter*	194	+	−
*Helicobacteraceae*	*Helicobacter*	209	−	+
*Nautiliaceae*	*Caminibacter*	191301	+	−
*Calotrichaceae*	*Calothrix*	1186	+	−
*Microcoleaceae*	*Planktothrix*	54304	+	−
*Deinococcaceae*	*Deinococcus*	1298	+	+
*Thermaceae*	*Allomeiothermus*	2935559	+	−
	*Meiothermus*	2747271	+	−
	*Oceanithermus*	208447	+	−
	*Thermus*	270	+	−
*Fusobacteriaceae*	*Fusobacterium*	848	+	−
*Leptotrichiaceae*	*Leptotrichia*	32067	+	+
	*Pseudoleptotrichia*	2755140	+	−
	*Sebaldella*	32068	+	+
	*Streptobacillus*	34104	+	−
*Gemmatimonadaceae*	*Gemmatimonas*	173479	−	+
*Mycoplasmataceae*	*Mycoplasmopsis*	2767358	+	−
*Archangiaceae*	*Archangium*	47	+	+
	*Cystobacter*	42	+	+
	*Melittangium*	44	+	+
	*Stigmatella*	40	+	+
*Myxococcaceae*	*Corallococcus*	83461	+	+
	*Myxococcus*	32	+	+
*Polyangiaceae*	*Chondromyces*	50	+	−
	*Polyangium*	55	+	+
	*Sorangium*	39643	+	+
*Nitrospiraceae*	*Nitrospira*	1234	+	−
*Aurantimonadaceae*	*Aurantimonas*	182269	+	−
	*Aureimonas*	414371	+	+
	*Jiella*	1775688	+	−
	*Martelella*	293088	+	+
*Boseaceae*	*Bosea*	85413	−	+
*Brucellaceae*	*Pseudochrobactrum*	354349	+	−
*Devosiaceae*	*Devosia*	46913	+	+
	*Paradevosia*	1573407	+	+
	*Pelagibacterium*	1082930	+	+
	*Youhaiella*	1827478	+	+
*Methylobacteriaceae*	*Microvirga*	186650	+	+
*Nitrobacteraceae*	*Bradyrhizobium*	374	+	−
	*Rhodopseudomonas*	1073	+	−
*Phyllobacteriaceae*	*Aquibium*	2911176	+	+
	*Mesorhizobium*	68287	+	+
	*Nitratireductor*	245876	+	+
	*Oricola*	1594166	+	+
	*Phyllobacterium*	28100	+	+
	*Roseitalea*	1915401	+	−
	*Salaquimonas*	2712688	+	−
*Rhizobiaceae*	*Agrobacterium*	357	+	+
	*Allorhizobium*	78526	+	+
	*Ciceribacter*	1648508	+	+
	*Ensifer*	106591	+	+
	*Georhizobium*	2661800	+	+
	*Neorhizobium*	1525371	+	+
	*Peteryoungia*	2853332	+	+
	*Pseudorhizobium*	1903858	+	+
	*Rhizobium*	379	+	+
	*Shinella*	323620	+	+
	*Sinorhizobium*	28105	+	+
*Stappiaceae*	*Labrenzia*	478070	+	+
	*Pannonibacter*	227873	+	+
	*Roseibium*	150830	+	+
	*Stappia*	152161	+	−
*Paracoccaceae*	*Algicella*	3050722	+	+
	*Cereibacter*	1653176	+	+
	*Frigidibacter*	1775705	+	+
	*Gemmobacter*	204456	+	+
	*Gymnodinialimonas*	2937410	+	+
	*Neotabrizicola*	2946607	−	+
	*Pacificitalea*	2846749	+	+
	*Paracoccus*	265	+	+
	*Parasedimentitalea*	2738399	+	+
	*Paroceanicella*	2683599	+	+
	*Pelagovum*	2795377	+	+
	*Polymorphum*	991903	+	−
	*Pontivivens*	1844015	+	+
	*Profundibacter*	2778525	−	+
	*Pseudooceanicola*	1679449	+	+
	*Pseudopuniceibacterium*	2613960	+	+
	*Pseudorhodobacter*	238783	−	+
	*Pukyongiella*	2831925	+	+
	*Qingshengfaniella*	2816884	+	+
	*Rhodobaca*	119541	+	+
	*Rhodovulum*	34008	+	+
	*Roseicitreum*	1209946	+	+
	*Silicimonas*	1955420	+	+
	*Tabrizicola*	1443919	+	+
	*Thioclava*	285107	−	+
*Roseobacteraceae*	*Celeribacter*	875170	+	+
	*Dinoroseobacter*	309512	+	+
	*Falsihalocynthiibacter*	2854182	+	+
	*Leisingera*	191028	+	+
	*Octadecabacter*	53945	+	+
	*Phaeobacter*	302485	+	+
	*Roseibacterium*	159345	+	+
	*Roseobacter*	2433	+	+
	*Roseovarius*	74030	+	+
	*Ruegeria*	97050	+	+
	*Salipiger*	263377	+	+
	*Sulfitobacter*	60136	+	+
*Acetobacteraceae*	*Acetobacter*	434	−	+
	*Lichenicola*	2804525	−	+
	*Roseomonas*	125216	+	+
*Azospirillaceae*	*Skermanella*	204447	+	+
*Thalassospiraceae*	*Thalassospira*	168934	+	+
*Erythrobacteraceae*	*Altererythrobacter*	361177	+	−
	*Aurantiacibacter*	2800681	+	−
	*Croceicoccus*	1295327	+	−
	*Pelagerythrobacter*	2800685	+	−
	*Qipengyuania*	1855416	+	−
	*Tsuneonella*	2800686	+	−
*Sphingomonadaceae*	*Novosphingobium*	165696	+	−
	*Sphingomonas*	13687	+	−
*Sphingosinicellaceae*	*Sphingosinicella*	335405	+	−
*Alcaligenaceae*	*Achromobacter*	222	+	+
	*Bordetella*	517	+	+
*Burkholderiaceae*	*Burkholderia*	32008	−	+
	*Chitinimonas*	240411	+	−
	*Cupriavidus*	106589	+	+
	*Ephemeroptericola*	2680021	+	−
	*Paraburkholderia*	1822464	−	+
	*Ralstonia*	48736	+	+
*Comamonadaceae*	*Acidovorax*	12916	+	+
	*Comamonas*	283	+	+
	*Delftia*	80865	+	+
	*Diaphorobacter*	238749	+	+
	*Ottowia*	219181	+	+
	*Paenacidovorax*	3051138	+	+
	*Paracidovorax*	3051137	+	+
	*Rhodoferax*	28065	+	+
*Oxalobacteraceae*	*Collimonas*	202907	+	+
	*Duganella*	75654	+	+
	*Janthinobacterium*	29580	+	+
	*Massilia*	149698	+	+
	*Pseudoduganella*	1522432	+	+
	*Telluria*	34069	+	+
	*Undibacterium*	401469	+	+
*Sphaerotilaceae*	*Caldimonas*	196013	+	−
	*Ideonella*	36862	+	−
	*Inhella*	644355	+	+
*Sutterellaceae*	*Sutterella*	40544	+	−
*Chromobacteriaceae*	*Chitinibacter*	230666	+	−
	*Chromobacterium*	535	+	+
	*Deefgea*	400947	+	−
	*Iodobacter*	32014	+	+
	*Paludibacterium*	400060	+	+
*Neisseriaceae*	*Alysiella*	194195	+	+
	*Chitinolyticbacter*	1055692	+	−
	*Conchiformibius*	334107	+	−
	*Kingella*	32257	+	+
	*Neisseria*	482	+	−
	*Simonsiella*	71	−	+
	*Wielerella*	2944815	−	+
*Thiobacillaceae*	*Thiobacillus*	919	+	−
*Azonexaceae*	*Dechloromonas*	73029	+	−
	*Ferribacterium*	88875	+	−
*Rhodocyclaceae*	*Niveibacterium*	1769726	+	+
*Zoogloeaceae*	*Azoarcus*	12960	+	−
	*Nitrogeniibacter*	2891294	+	−
*Aeromonadaceae*	*Aeromonas*	642	+	+
*Alteromonadaceae*	*Agarivorans*	261825	+	+
	*Hydrocarboniclastica*	2650549	+	−
	*Saliniradius*	2661818	+	−
*Colwelliaceae*	*Litorilituus*	1407056	+	−
*Ferrimonadaceae*	*Ferrimonas*	44011	+	+
*Idiomarinaceae*	*Idiomarina*	135575	+	+
*Pseudoalteromonadaceae*	*Pseudoalteromonas*	53246	+	+
	*Psychrosphaera*	907197	−	+
*Psychromonadaceae*	*Psychromonas*	67572	+	+
*Shewanellaceae*	*Parashewanella*	2547964	+	+
	*Shewanella*	22	+	+
*Cellvibrionaceae*	*Marinagarivorans*	1792291	−	+
	*Saccharophagus*	316625	−	+
	*Teredinibacter*	2425	+	+
*Microbulbiferaceae*	*Microbulbifer*	48073	+	+
*Spongiibacteraceae*	*Spongiibacter*	630749	+	−
*Chromatiaceae*	*Allochromatium*	85072	+	−
	*Caldichromatium*	2828366	+	−
	*Marichromatium*	85076	+	−
	*Thermochromatium*	85073	+	−
	*Thiocapsa*	1056	+	−
	*Thiocystis*	13724	+	−
*Ectothiorhodospiraceae*	*Thioalkalivibrio*	106633	+	−
*Granulosicoccaceae*	*Granulosicoccus*	437504	+	+
*Budviciaceae*	*Pragia*	82984	−	+
*Enterobacteriaceae*	*Atlantibacter*	1903434	+	+
	*Cedecea*	158483	+	+
	*Citrobacter*	544	+	+
	*Cronobacter*	413496	+	+
	*Enterobacter*	547	+	+
	*Escherichia*	561	+	+
	*Jejubacter*	2815296	+	+
	*Klebsiella*	570	+	+
	*Kluyvera*	579	+	+
	*Kosakonia*	1330547	+	+
	*Leclercia*	83654	+	+
	*Lelliottia*	1330545	+	+
	*Plesiomonas*	702	+	+
	*Pseudocitrobacter*	1504576	+	+
	*Raoultella*	160674	+	+
	*Salmonella*	590	+	+
	*Scandinavium*	2726810	+	+
	*Shigella*	620	+	+
	*Shimwellia*	1335483	+	−
	*Symbiopectobacterium*	801	+	+
*Erwiniaceae*	*Erwinia*	551	+	+
	*Mixta*	2100764	+	+
	*Pantoea*	53335	+	+
*Hafniaceae*	*Edwardsiella*	635	+	−
	*Hafnia*	568	+	+
	*Obesumbacterium*	82982	+	+
*Morganellaceae*	*Arsenophonus*	637	+	−
	*Morganella*	581	+	+
	*Photorhabdus*	29487	+	+
	*Proteus*	583	+	+
	*Providencia*	586	+	+
	*Xenorhabdus*	626	+	+
*Pectobacteriaceae*	*Brenneria*	71655	+	+
	*Dickeya*	204037	+	+
*Pectobacteriaceae*	*Pectobacterium*	122277	+	+
*Yersiniaceae*	*Chania*	1745211	+	+
	*Gibbsiella*	929812	+	−
	*Rouxiella*	1565532	−	+
	*Serratia*	613	+	+
	*Yersinia*	629	+	+
*Legionellaceae*	*Legionella*	445	+	−
*Methylococcaceae*	*Methylocaldum*	73778	+	−
	*Methylococcus*	413	+	−
	*Methylomagnum*	1760987	+	−
	*Methylomonas*	416	+	−
*Moraxellaceae*	*Acinetobacter*	469	+	+
	*Aquirhabdus*	2824158	−	+
	*Moraxella*	475	−	+
*Nevskiaceae*	*Solimonas*	413435	+	–
*Alcanivoracaceae*	*Alcanivorax*	59753	+	+
	*Alloalcanivorax*	3020832	+	+
*Endozoicomonadaceae*	*Endozoicomonas*	305899	+	+
*Halomonadaceae*	*Cobetia*	204286	+	+
	*Zymobacter*	33073	+	−
*Oceanospirillaceae*	*Aliamphritea*	3018276	+	−
	*Marinomonas*	28253	+	+
	*Neptunomonas*	75687	+	−
	*Thalassolituus*	187492	+	+
*Saccharospirillaceae*	*Gynuella*	1445504	+	+
	*Reinekea*	230494	+	−
	*Saccharospirillum*	231683	+	+
*Orbaceae*	*Frischella*	1335631	−	+
	*Gilliamella*	1193503	+	+
	*Zophobihabitans*	2894762	+	−
*Pasteurellaceae*	*Actinobacillus*	713	+	+
	*Aggregatibacter*	416916	+	+
	*Avibacterium*	292486	+	+
	*Basfia*	697331	+	+
	*Bisgaardia*	109471	+	+
	*Frederiksenia*	1649317	+	+
	*Haemophilus*	724	+	+
	*Mannheimia*	75984	+	+
	*Otariodibacter*	1249016	+	+
	*Pasteurella*	745	+	+
	*Rodentibacter*	1960084	+	+
*Pseudomonadaceae*	*Pseudomonas*	286	+	−
	*Stutzerimonas*	2901164	+	−
*Piscirickettsiaceae*	*Thiomicrorhabdus*	2039723	+	−
*Thiotrichaceae*	*Beggiatoa*	1021	+	−
	*Thiothrix*	1030	+	+
*Vibrionaceae*	*Aliivibrio*	511678	+	+
	*Paraphotobacterium*	2042066	+	−
	*Photobacterium*	657	+	+
	*Salinivibrio*	51366	+	+
	*Thaumasiovibrio*	2014233	+	−
	*Vibrio*	662	+	+
*Rhodanobacteraceae*	*Aerosticca*	2707020	−	+
	*Dokdonella*	323413	−	+
	*Dyella*	231454	−	+
	*Frateuria*	70411	−	+
	*Luteibacter*	242605	−	+
	*Rhodanobacter*	75309	−	+
*Xanthomonadaceae*	*Pseudolysobacter*	2709666	−	+
*Brachyspiraceae*	*Brachyspira*	29521	+	−
*Breznakiellaceae*	*Breznakiella*	2845254	+	+
	*Gracilinema*	2951106	+	+
	*Leadbettera*	2951107	+	−
*Sphaerochaetaceae*	*Sphaerochaeta*	399320	+	+
*Spirochaetaceae*	*Entomospira*	2834378	+	+
	*Sediminispirochaeta*	1911556	+	−
	*Thiospirochaeta*	2792240	+	+
*Treponemataceae*	*Brucepastera*	2967962	+	−
	*Treponema*	157	+	+
*Synergistaceae*	*Thermanaerovibrio*	81461	+	−
*Desulfobacteraceae*	*Desulforapulum*	2904687	+	−
*Desulfosarcinaceae*	*Desulfosarcina*	2299	+	−
*Desulfobulbaceae*	*Desulfobulbus*	893	+	−
*Desulfocapsaceae*	*Desulfosediminicola*	2886823	+	−
*Desulfomicrobiaceae*	*Desulfomicrobium*	898	+	−
*Desulfovibrionaceae*	*Desulfovibrio*	872	+	−
	*Pseudodesulfovibrio*	2035811	+	−
	*Salidesulfovibrio*	2950010	+	−
	*Solidesulfovibrio*	2910984	+	−
*Desulfuromonadaceae*	*Pelobacter*	18	+	−
*Geobacteraceae*	*Trichlorobacter*	115782	+	−
*Syntrophobacteraceae*	*Syntrophobacter*	29526	+	−
*Petrotogaceae*	*Defluviitoga*	1511648	+	+
	*Marinitoga*	160798	+	+
	*Oceanotoga*	1255275	+	+
	*Petrotoga*	28236	+	+
	*Tepiditoga*	2778400	+	+
*Fervidobacteriaceae*	*Fervidobacterium*	2422	+	+
	*Thermosipho*	2420	+	+
*Opitutaceae*	*Horticoccus*	2986286	−	+
	*Opitutus*	178440	−	+

^1^ Genera highlighted in red are not complete according to Section 3.2.1.

**Table 8:** Presence (+) or absence (−) of *ushA*-like (U) and *cpdB*-like (C) genes in selected bacterial species. The (+) background indicates: green, presence in ≤50% of the genomes analyzed; orange, presence in >50% but <100% of the genomes; red, presence in all the genomes. Full data can be found in Table S6.

Species ^1^	Taxid	U	C
*Acinetobacter calcoaceticus*	471	+	−
*Aerococcus urinae*	1376	−	−
*Aeromonas hydrophila*	644	+	+
*Bacillus anthracis*	1392	+	+
*Bacillus cereus*	1396	+	+
*Bacillus subtilis*	1423	+	+
*Borrelia burgdorferi*	139	−	−
*Brucella abortus*	235	−	−
*Brucella melitensis*	29459	−	−
*Brucella suis*	29461	−	−
*Campylobacter jejuni*	197	−	−
*Chlamydia abortus*	83555	−	−
*Chlamydia muridarum*	83560	−	−
*Chlamydia pecorum*	85991	−	−
*Chlamydia pneumoniae*	83558	−	−
*Chlamydia psittaci*	83554	−	−
*Chlamydia trachomatis*	813	−	−
*Citrobacter freundii*	546	+	+
*Citrobacter koseri*	545	+	+
*Citrobacter rodentium*	67825	+	+
*Clostridioides difficile*	1496	+	−
*Clostridium botulinum*	1491	+	+
*Clostridium perfringens*	1502	+	+
*Clostridium tetani*	1513	−	+
*Corynebacterium diphtheriae*	1717	−	−
*Coxiella burnetii*	777	−	−
*Enterobacter cloacae*	550	+	+
*Enterococcus avium*	33945	+	+
*Enterococcus faecalis*	1351	+	+
*Enterococcus faecium*	1352	+	+
*Escherichia albertii*	208962	+	+
*Escherichia coli*	562	+	+
*Escherichia fergusonii*	564	+	+
*Francisella tularensis*	263	−	−
*Haemophilus influenzae*	727	+	+
*Haemophilus parainfluenzae*	729	+	+
*Hafnia alvei*	569	+	+
*Helicobacter pylori*	210	−	+
*Klebsiella aerogenes*	548	+	+
*Klebsiella oxytoca*	571	+	+
*Klebsiella pneumoniae*	573	+	+
*Kluyvera ascorbate*	51288	+	+
*Legionella pneumophila*	446	+	−
*Leptospira* *borgpetersenii*	174	−	−
*Leptospira interrogans*	173	−	−
*Leptospira kirschneri*	29507	−	−
*Leptospira noguchii*	28182	−	−
*Leptospira santarosai*	28183	−	−
*Leptospira weilii*	28184	−	−
*Listeria monocytogenes*	1639	+	−
*Moraxella catarrhalis*	480	−	−
*Morganella morganii*	582	+	+
*Mycobacterium avium*	1764	−	−
*Mycobacterium intracellulare*	1767	−	−
*Mycobacterium leprae*	1769	−	−
*Mycobacterium tuberculosis*	1773	−	−
*Mycobacterium ulcerans*	1809	−	−
*Mycoplasma leachii*	2105	−	−
*Mycoplasma mycoides*	2102	−	−
*Mycoplasma putrefaciens*	2123	−	−
*Neisseria gonorrhoeae*	485	−	−
*Neisseria meningitidis*	487	−	−
*Pasteurella multocida*	747	+	+
*Plesiomonas shigelloides*	703	+	+
*Proteus mirabilis*	584	+	+
*Proteus vulgaris*	585	+	+
*Providencia stuartii*	588	+	+
*Pseudomonas aeruginosa*	287	−	−
*Pseudomonas fluorescens*	294	+	−
*Rickettsia rickettsii*	783	−	−
*Salmonella bongori*	54736	+	+
*Salmonella enterica*	28901	+	+
*Salmonella enterica* subsp. *arizonae*	59203	+	+
*Salmonella enterica* subsp. *diarizonae*	59204	+	+
*S. enterica* subsp. *enterica* ser. Pullorum	605	+	+
*S. enterica* subsp. *enterica* ser. Typhi	90370	+	+
*S. enterica* sub. *enterica* ser. Typhimurium	90371	−	+
*Salmonella enterica* subsp. *houtenae*	59205	+	+
*Salmonella enterica* subsp. *salamae*	59202	+	+
*Salmonella enterica* subsp. *VII*	59208	+	+
*Serratia liquefaciens*	614	+	+
*Serratia marcescens*	615	+	+
*Shigella boydii*	621	+	+
*Shigella dysenteriae*	622	+	+
*Shigella flexneri*	623	+	+
*Shigella sonnei*	624	+	+
*Staphylococcus aureus*	1280	+	−
*Staphylococcus epidermidis*	1282	+	−
*Staphylococcus saprophyticus*	29385	+	+
*Staphylococcus warnerii *	1292	+	+
*Stenotrophomonas maltophilia*	40324	−	−
*Streptococcus agalactiae*	1311	+	+
*Streptococcus dysgalactiae*	1334	+	+
*Streptococcus mitis*	28037	−	−
*Streptococcus mutans*	1309	+	−
*Streptococcus parasuis*	1501662	+	+
*Streptococcus pneumoniae*	1313	−	−
*Streptococcus pyogenes*	1314	+	−
*Streptococcus sanguinis*	1305	+	+
*Streptococcus suis*	1307	+	+
*Streptococcus thermophilus*	1308	+	+
*Treponema pallidum*	160	+	−
*Vibrio cholerae*	666	+	+
*Yersinia enterocolitica*	630	+	+
*Yersinia intermedia*	631	+	+
*Yersinia pestis*	632	+	+
*Yersinia pseudotuberculosis*	633	+	+

^1^ Species highlighted in red are not complete according to Section 3.2.1.

**Table 9:** Numbers of taxa probed, analyzed, and deemed complete after TBlastN analyses with UshA-like (U) and CpdB-like (C) probes: breakdown by kind of results obtained, with presence (+) and/or absence (−) of hits with each probe type. The data are computed from the tables indicated.

Level	(Tables)	Number of Taxa Probed ^1^	Number of Taxa without Genomes ^2^	Number of Taxa Analyzed ^3^	Number of Complete Taxa ^4^	Breakdown of Complete Taxa by Kind of Results			
						U+ C+	U+ C−	U− C+	U− C−
Phylum	Table 3 and Table S1	43	0	43	19	0	2	3	14
Class	Table 4 and Table S2	76	6	70	31	1	6	0	24
Order	Table 5 and Table S3	152	20	132	53	4	4	2	43
Family	Table 6 and Table S4	403	99	304	139	7	16	3	113
Genus	Table 7 and Table S5	510	0	510	268	136	95	37	0
Species	Table 8 and Table S6	107	0	107	80	37	5	1	37
Total	-	1291	125	1166	590	185	128	46	231

^1^ Taxa that were submitted to TBlastN analysis with the seven probes. ^2^ Taxa that, according to TBlastN analysis, do not contain sequenced genomes in the NCBI Complete Genomes Database. ^3^ Taxa with sequenced genomes in the NCBI Complete Genomes Database, and that were effectively analyzed. ^4^ Taxa declared complete according to Section 3.2.1: in Table 3, Table 4, Table 5, Table 6, Table 7 and Table 8, the names of non-complete taxa are written in red type.

**Table 10:** Complete taxa that contain both *ushA*-like and *cpdB*-like genes. No bacterial phylum showed these characteristics.

**Class**		**Genus**		**Genus**	
*Limnochordia*	1676648	*Kitasatospora*	2063	*Roseibium*	150830
**Order**		*Kluyvera*	579	*Roseicitreum*	1209946
*Egicoccales*	1755823	*Kosakonia*	1330547	*Roseobacter*	2433
*Kosmotogales*	1643946	*Kroppenstedtia*	1274351	*Saccharibacillus*	456492
*Mesoaciditogales*	1769716	*Labrenzia*	478070	*Saccharospirillum*	231683
*Nannocystales*	3031713	*Leclercia*	83654	*Scandinavium*	2726810
**Family**		*Leisingera*	191028	*Sebaldella*	32068
*Breoghaniaceae*	2831104	*Lentilactobacillus*	2767893	*Sediminibacillus*	482460
*Devosiaceae*	2831106	*Lentzea*	165301	*Silicimonas*	1955420
*Kiloniellaceae*	597359	*Luteipulveratus*	745364	*Sporolactobacillus*	2077
*Kribbellaceae*	2726069	*Mangrovibacillus*	2920444	*Stigmatella*	40
*Moritellaceae*	267891	*Marinomonas*	28253	*Streptosporangium*	2000
*Oleiphilaceae*	191033	*Martelella*	293088	*Sutcliffiella*	2837511
*Thermosediminibacteraceae*	2770093	*Melittangium*	44	*Symbiopectobacterium*	801
**Genus**		*Metabacillus*	2675233	*Tabrizicola*	1443919
*Acetilactobacillus*	2767874	*Methylomusa*	2093783	*Telluria*	34069
*Actinoplanes*	1865	*Mixta*	2100764	*Tepiditoga*	2778400
*Algicella*	3050722	*Morganella*	581	*Thalassospira*	168934
*Alloalcanivorax*	3020832	*Neorhizobium*	1525371	*Thermosipho*	2420
*Allobacillus*	1400133	*Nicoliella*	2978367	*Thiospirochaeta*	2792240
*Amylolactobacillus*	2767876	*Nitratireductor*	245876	*Tissierella*	41273
*Anaerotruncus*	244127	*Niveibacterium*	1769726	*Weizmannia*	2817139
*Apilactobacillus*	2767877	*Obesumbacterium*	82982	*Xenorhabdus*	626
*Aquibium*	2911176	*Occultella*	2828348	*Yersinia*	629
*Atlantibacter*	1903434	*Oceanotoga*	1255275	*Youhaiella*	1827478
*Basfia*	697331	*Oricola*	1594166	**Species**	
*Bisgaardia*	109471	*Otariodibacter*	1249016	*Aeromonas hydrophila*	644
*Breznakiella*	2845254	*Pacificitalea*	2846749	*Bacillus anthracis*	1392
*Carboxydothermus*	129957	*Paenacidovorax*	3051138	*Bacillus cereus*	1396
*Cedecea*	158483	*Paenalkalicoccus*	2944627	*Citrobacter freundii*	546
*Celeribacter*	875170	*Pannonibacter*	227873	*Citrobacter koseri*	545
*Chania*	1745211	*Paracidovorax*	3051137	*Citrobacter rodentium*	67825
*Ciceribacter*	1648508	*Paradevosia*	1573407	*Enterobacter cloacae*	550
*Citrobacter*	544	*Paraliobacillus*	200903	*Escherichia albertii*	208962
*Cobetia*	204286	*Parasedimentitalea*	2738399	*Escherichia fergusonii*	564
*Cronobacter*	413496	*Parashewanella*	2547964	*Haemophilus influenzae*	727
*Cystobacter*	42	*Paroceanicella*	2683599	*H. parainfluenzae*	729
*Cytobacillus*	2675230	*Pelagibacterium*	1082930	*Hafnia alvei*	569
*Defluviitoga*	1511648	*Pelagovum*	2795377	*Klebsiella aerogenes*	548
*Dickeya*	204037	*Periweissella*	2930384	*Klebsiella oxytoca*	571
*Dinoroseobacter*	309512	*Peterkaempfera*	2995704	*Kluyvera ascorbata*	51288
*Duganella*	75654	*Peteryoungia*	2853332	*Morganella morganii*	582
*Entomospira*	2834378	*Petrocella*	2603323	*Proteus mirabilis*	584
*Falsihalocynthiibacter*	2854182	*Petrotoga*	28236	*Proteus vulgaris*	585
*Flavonifractor*	946234	*Phaeobacter*	302485	*Salmonella bongori*	54736
*Frederiksenia*	1649317	*Photorhabdus*	29487	*S. enterica* subsp. *arizonae*	59203
*Frigidibacter*	1775705	*Polyangium*	55	*S. enterica* subsp. *diarizonae*	59204
*Fructilactobacillus*	2767881	*Polycladomyces*	1348505	*S. enterica* subsp. *enterica ser. Pullorum*	605
*Fructobacillus*	559173	*Pontivivens*	1844015	*S. enterica* subsp. *enterica ser. Typhi*	90370
*Georhizobium*	2661800	*Priestia*	2800373	*S. enterica* subsp. *houtenae*	59205
*Gilliamella*	1193503	*Proteus*	583	*S. enterica* subsp. *salamae*	59202
*Gracilibacillus*	74385	*Pseudocitrobacter*	1504576	*S. enterica* subsp. *VII*	59208
*Gracilinema*	2951106	*Pseudoduganella*	1522432	*Serratia liquefaciens*	614
*Granulosicoccus*	437504	*Pseudooceanicola*	1679449	*Shigella boydii*	621
*Gymnodinialimonas*	2937410	*Pseudopuniceibacterium*	2613960	*Shigella dysenteriae*	622
*Gynuella*	1445504	*Pseudorhizobium*	1903858	*Shigella flexneri*	623
*Halobacteroides*	42417	*Psychrobacillus*	1221880	*Shigella sonnei*	624
*Hafnia*	568	*Pukyongiella*	2831925	*Streptococcus sanguinis*	1305
*Halocella*	46466	*Qingshengfaniella*	2816884	*Streptococcus thermophilus*	1308
*Inhella*	644355	*Radiobacillus*	2785518	*Yersinia enterocolitica*	630
*Intrasporangium*	53357	*Rhodobaca*	119541	*Yersinia intermedia*	631
*Iocasia*	2899804	*Rhodovulum*	34008	*Yersinia pestis*	632
*Jejubacter*	2815296	*Roseibacterium*	159345	*Yersinia pseudotuberculosis*	633

**Table 11:** Complete taxa that do not contain *ushA*-like or *cpdB*-like genes. No complete genus showed these characteristics.

**Phylum**		**Order**		**Family**	
*Abditibacteriota*	2109258	*Synechococcales*	1890424	*Paludibacteraceae*	2005523
*Acidobacteriota*	57723	*Thermoleophilales*	588674	*Parvibaculaceae*	2813035
*Aquificota*	200783	*Thermostichales*	2881383	*Peptococcaceae*	186807
*Bdellovibrionota*	3018035	*Trueperales*	2762275	*Peptoniphilaceae*	1570339
*Chlamydiota*	204428	**Family**		*Phreatobacteraceae*	2843305
*Chlorobiota*	1090	*Acetomicrobiaceae*	3029086	*Porticoccaceae*	1706374
*Chrysiogenota*	200938	*Acidilutibacteraceae*	2992717	*Proteinivoraceae*	1491775
*Elusimicrobiota*	74152	*Acidimicrobiaceae*	84994	*Rhodospirillaceae*	41295
*Kiritimatiellota*	134625	*Aminobacteriaceae*	3029087	*Rikenellaceae*	171550
*Lentisphaerota*	256845	*Amorphaceae*	2685818	*Rivulariaceae*	1185
*Nitrospinota*	1293497	*Anaeromyxobacteraceae*	1524215	*Salinivirgaceae*	1970190
*Planctomycetota*	203682	*Aphanizomenonaceae*	1892259	*Sandaracinaceae*	1055686
*Rhodothermota*	1853220	*Aristaeellaceae*	3046368	*Segniliparaceae*	316606
*Thermomicrobiota*	3027942	*Bartonellaceae*	772	*Stellaceae*	2844601
**Class**		*Beijerinckiaceae*	45404	*Steroidobacteraceae*	2689614
*Acidithiobacillia*	1807140	*Beutenbergiaceae*	125316	*Sterolibacteriaceae*	2008793
*Anaerolineae*	292625	*Blastochloridaceae*	2831090	*Succinivibrionaceae*	83763
*Armatimonadia*	1042312	*Borreliaceae*	1643685	*Sulfuricellaceae*	2772226
*Caldilineae*	475962	*Bruguierivoracaceae*	2812006	*Sulfurimonadaceae*	2771471
*Chitinophagia*	1853228	*Calditerrivibrionaceae*	2945021	*Sulfurospirillaceae*	2932623
*Coriobacteriia*	84998	*Cardiobacteriaceae*	868	*Sulfurovaceae*	2771472
*Cytophagia*	768503	*Cellulomonadaceae*	85016	*Symbiobacteriaceae*	543349
*Desulfarculia*	3031646	*Cellulosilyticaceae*	3018741	*Syntrophomonadaceae*	68298
*Desulfobaccia*	3031647	*Chelatococcaceae*	2036754	*Syntrophotaleaceae*	2812024
*Desulfurellia*	3031853	*Christensenellaceae*	990719	*Tenuifilaceae*	2760872
*Fibrobacteria*	204430	*Coleofasciculaceae*	1892251	*Tepidanaerobacteraceae*	2770092
*Flavobacteriia*	117743	*Coprobacillaceae*	2810280	*Terasakiellaceae*	2813951
*Hydrogenophilalia*	2008785	*Coxiellaceae*	118968	*Thalassobaculaceae*	2844864
*Ktedonobacteria*	388447	*Deferribacteraceae*	191394	*Thermincolaceae*	2937911
*Longimicrobiia*	1804991	*Demequinaceae*	1042322	*Thermodesulfobacteriaceae*	188711
*Methylacidiphilae*	1955630	*Desulfallaceae*	2867375	*Thermodesulfobiaceae*	227387
*Mycoplasmoidales*	2790996	*Desulfococcaceae*	2931039	*Thermovirgaceae*	3029089
*Saprospiria*	1937959	*Desulfohalobiaceae*	213117	*Thioalkalibacteraceae*	2035710
*Spartobacteria*	134549	*Desulfolunaceae*	3031622	*Thioalkalispiraceae*	1096778
*Sphingobacteriia*	117747	*Desulfosudaceae*	2904715	*Tolypothrichaceae*	119859
*Tepidiformia*	2682225	*Desulfotomaculaceae*	2937910	*Tropherymataceae*	2805591
*Thermodesulfovibrionia*	2811502	*Dethiosulfovibrionaceae*	3029088	*Wenzhouxiangellaceae*	1676141
*Verrucomicrobiae*	203494	*Dietziaceae*	85029	*Woeseiaceae*	1738654
*Zetaproteobacteria*	580370	*Dysgonomonadaceae*	2005520	*Xanthobacteraceae*	335928
**Order**		*Fastidiosibacteraceae*	2056687	*Zymomonadaceae*	2844881
*Acholeplasmatales*	186329	*Fimbriimonadaceae*	1663426	**Species**	
*Acidaminococcales*	1843488	*Flexistipitaceae*	2945022	*Aerococcus urinae*	1376
*Acidiferrobacterales*	1692040	*Fluviibacteraceae*	2808923	*Borrelia burgdorferi*	139
*Acidothermales*	1643683	*Francisellaceae*	34064	*Brucella abortus*	235
*Actinopolysporales*	622450	*Gallionellaceae*	90627	*Brucella melitensis*	29459
*Bifidobacteriales*	85004	*Geminicoccaceae*	2066434	*Brucella suis*	29461
*Brevinematales*	1643687	*Geoalkalibacteraceae*	3031665	*Campylobacter jejuni*	197
*Catenulisporales*	414714	*Gomontiellaceae*	1892255	*Chlamydia abortus*	83555
*Caulobacterales*	204458	*Gordoniaceae*	85026	*Chlamydia muridarum*	83560
*Chroococcidiopsidales*	1890505	*Halarsenatibacteraceae*	3046411	*Chlamydia pecorum*	85991
*Chroococcales*	1118	*Halieaceae*	1706372	*Chlamydia pneumoniae*	83558
*Dehalococcoidales*	1202465	*Halothermotrichaceae*	3046412	*Chlamydia psittaci*	83554
*Dehalogenimonas*	670486	*Halothiobacillaceae*	255526	*Chlamydia trachomatis*	813
*Egibacterales*	1747768	*Hapalosiphonaceae*	1892263	*Corynebacterium diphtheriae*	1717
*Emcibacterales*	2066490	*Heliobacteriaceae*	31984	*Coxiella burnetiid*	777
*Entomoplasmatales*	186328	*Hoyosellaceae*	3040680	*Francisella tularensis*	263
*Euzebyales*	908621	*Hydrogenimonadaceae*	292630	*Leptospira borgpetersenii*	174
*Ferrovales*	1442155	*Hyphomicrobiaceae*	45401	*Leptospira interrogans*	173
*Frankiales*	85013	*Iamiaceae*	633392	*Leptospira kirschneri*	29507
*Gloeobacterales*	307595	*Jonesiaceae*	85022	*Leptospira noguchii*	28182
*Holosporales*	1921002	*Kaistiaceae*	2831111	*Leptospira santarosai*	28183
*Kangiellales*	2887327	*Labilitrichaceae*	1524216	*Leptospira weilii*	28184
*Koleobacterales*	2786987	*Lawsonellaceae*	2805586	*Moraxella catarrhalis*	480
*Kordiimonadales*	362534	*Lichenihabitantaceae*	2723775	*Mycobacterium avium*	1764
*Leptospirales*	1643688	*Litorivicinaceae*	449732	*Mycobacterium intracellulare*	1767
*Magnetococcales*	1191478	*Maliibacteriaceae*	3047432	*Mycobacterium leprae*	1769
*Maricaulales*	2800059	*Marinobacteraceae*	2887365	*Mycobacterium tuberculosis*	1773
*Marinilabiliales*	1970189	*Melioribacteraceae*	1334117	*Mycobacterium ulcerans*	1809
*Minwuiales*	2493627	*Methylocystaceae*	31993	*Mycoplasma leachii*	2105
*Moorellales*	3039167	*Methylophilaceae*	32011	*Mycoplasma mycoides*	2102
*Nakamurellales*	1643684	*Methylothermaceae*	1486721	*Mycoplasma putrefaciens*	2123
*Natranaerobiales*	485256	*Mucispirillaceae*	2945020	*Neisseria gonorrhoeae*	485
*Parvularculales*	255473	*Mycobacteriaceae*	1762	*Neisseria meningitidis*	487
*Pleurocapsales*	52604	*Nitratiruptoraceae*	2795691	*Pseudomonas aeruginosa*	287
*Pseudanabaenales*	2881377	*Nitrosomonadaceae*	206379	*Rickettsia rickettsii*	783
*Puniceicoccales*	415001	*Nocardiopsaceae*	83676	*Stenotrophomonas maltophilia*	40324
*Rickettsiales*	766	*Nostocaceae*	1162	*Streptococcus mitis*	28037
*Sneathiellales*	510684	*Oscillatoriaceae*	1892254	*Streptococcus pneumoniae*	1313
*Solirubrobacterales*	588673				

**Table 12:** Complete taxa that contain *ushA*-like but not *cpdB*-like genes.

**Phylum**		**Genus**		**Genus**	
*Atribacterota*	67818	*Beggiatoa*	1021	*Pelagerythrobacter*	2800685
*Caldisericota*	67814	*Brucepastera*	2967962	*Pelobacter*	18
**Class**		*Caldichromatium*	2828366	*Phytohabitans*	907364
*Chloroflexia*	32061	*Chitinimonas*	240411	*Planktothrix*	54304
*Chthonomonadetes*	1077257	*Chitinolyticbacter*	1055692	*Polymorphum*	991903
*Desulfomonilia*	3031650	*Chondromyces*	50	*Pseudochrobactrum*	354349
*Dictyoglomia*	203486	*Clostridioides*	1870884	*Pseudoleptotrichia*	2755140
*Syntrophia*	3031648	*Companilactobacillus*	2767879	*Pullulanibacillus*	475230
*Thermoflexia*	1495646	*Conchiformibius*	334107	*Reinekea*	230494
**Order**		*Croceicoccus*	1295327	*Rhodopseudomonas*	1073
*Bradymonadales*	1779134	*Deefgea*	400947	*Roseitalea*	1915401
*Gloeomargaritales*	1955042	*Dermatophilus*	1862	*Salaquimonas*	2712688
*Glycomycetales*	85014	*Desulforapulum*	2904687	*Salidesulfovibrio*	2950010
*Immundisolibacterales*	1934945	*Edwardsiella*	635	*Salimicrobium*	351195
**Family**		*Effusibacillus*	1502725	*Saliniradius*	2661818
*Aminithiophilaceae*	3029085	*Ephemeroptericola*	2680021	*Salinispora*	168694
*Desulfatibacillaceae*	3031627	*Ferribacterium*	88875	*Sediminispirochaeta*	1911556
*Elioraeaceae*	2690195	*Fervidibacillus*	3033930	*Serinicoccus*	265976
*Geovibrionaceae*	2945019	*Geodermatophilus*	1860	*Shimwellia*	1335483
*Gottschalkiaceae*	2042895	*Geosporobacter*	390805	*Solidesulfovibrio*	2910984
*Hahellaceae*	224379	*Gibbsiella*	929812	*Solimonas*	413435
*Ilumatobacteraceae*	2448023	*Gudongella*	2692382	*Spongiibacter*	630749
*Kytococcaceae*	2805426	*Halalkalibacter*	2893056	*Staphylospora*	2689589
*Listeriaceae*	186820	*Hathewaya*	1769729	*Stappia*	152161
*Pleomorphomonadaceae*	2843308	*Hydrocarboniclastica*	2650549	*Suicoccus*	2689587
*Tepidimicrobiaceae*	2992719	*Ideonella*	36862	*Syntrophobacter*	29526
*Thermodesulfatatoraceae*	3031464	*Jeotgalibacillus*	157226	*Tetragenococcus*	51668
*Thermohalobacteraceae*	2848916	*Jiella*	1775688	*Thermanaerovibrio*	81461
*Thermotogaceae*	188709	*Kibdelosporangium*	2029	*Thermobacillus*	76632
*Usitatibacteraceae*	2803844	*Leadbettera*	2951107	*Thermobispora*	147067
*Zooshikellaceae*	2898533	*Lederbergia*	2804231	*Thermochromatium*	85073
**Genus**		*Litorilituus*	1407056	*Thermus*	270
*Aceticella*	3051499	*Marichromatium*	85076	*Thiocapsa*	1056
*Acetoanaerobium*	186831	*Meiothermus*	2747271	*Thiocystis*	13724
*Actinosynnema*	40566	*Methylocaldum*	73778	*Trichlorobacter*	115782
*Aliamphritea*	3018276	*Methylococcus*	413	*Tsuneonella*	2800686
*Allochromatium*	85072	*Methylomagnum*	1760987	*Tumebacillus*	432330
*Allomeiothermus*	2935559	*Modestobacter*	88138	*Zophobihabitans*	2894762
*Amphibacillus*	29331	*Nitrogeniibacter*	2891294	*Zymobacter*	33073
*Anaeropeptidivorans*	2997360	*Nosocomiicoccus*	489909	**Species**	
*Arsenicicoccus*	267408	*Novibacillus*	1677050	*Clostridioides difficile*	1496
*Aurantimonas*	182269	*Oceanithermus*	208447	*Staphylococcus epidermidis*	1282
*Austwickia*	1184606	*Odoribacter*	283168	*Streptococcus mutans*	1309
*Barnesiella*	397864	*Parageobacillus*	1906945	*Streptococcus pyogenes*	1314
				*Treponema pallidum*	160

**Table 13:** Complete taxa that contain *cpdB*-like but not *ushA*-like genes. There was no bacterial class showing these characteristics.

**Phylum**		**Genus**		**Genus**	
*Balneolota*	1936987	*Dokdonella*	323413	*Profundibacter*	2778525
*Calditrichota*	1930617	*Duncaniella*	2518495	*Pseudolysobacter*	2709666
*Coprothermobacterota*	2138240	*Dyella*	231454	*Pseudoprevotella*	2884814
**Order**		*Flaviflexus*	1522056	*Pseudorhodobacter*	238783
*Haliangiales*	3031714	*Frateuria*	70411	*Rhodanobacter*	75309
*Sporichthyales*	2495578	*Frischella*	1335631	*Rouxiella*	1565532
**Family**		*Gemmatimonas*	173479	*Saccharophagus*	316625
*Ignatzschineriaceae*	3018589	*Herbinix*	1663717	*Simonsiella*	71
*Ignavibacteriaceae*	795749	*Horticoccus*	2986286	*Sodaliphilus*	2815786
*Vulgatibacteraceae*	1524213	*Kineococcus*	33981	*Streptantibioticus*	2995706
**Genus**		*Lichenicola*	2804525	*Tetrasphaera*	99479
*Actinocatenispora*	390988	*Luteibacter*	242605	*Thioclava*	285107
*Aerosticca*	2707020	*Marinagarivorans*	1792291	*Tyzzerella*	1506577
*Anaeromicropila*	3024823	*Muribaculum*	1918540	*Wielerella*	2944815
*Aquirhabdus*	2824158	*Neotabrizicola*	2946607	**Species**	
*Caldanaerobacter*	249529	*Opitutus*	178440	*Clostridium tetani*	1513
*Dermabacter*	36739	*Pragia*	82984		

**Table 14:** Specicity of probe O32133 for the phylum *Bacillota*. Comparison between results obtained by querying type-material genomes of the phylum *Bacillota* and of Superkingdom *Bacteria* excluding *Bacillota*.

		Phylum *Bacillota* (Taxid:1239) (705 Type-Material Genomes)			Superkingdom *Bacteria* (Taxid:2) Excluding Phylum *Bacillota* (Taxid:1239) (3526 Type-Material Genomes)		
Type of Probe	Probe ^1^	Hits	Score Max.	Score Min.	Hits	Score Max.	Score Min.
UshA-like	P07024	120	235	151	521	1099	151
	P44569	77	328	151	395	1188	151
	WP_000726911	147	904	151	324	247	151
	WP_011837008	94	1297	151	19	213	151
	O32133	366	947	171	0 (4) *	(416) *	(175) *
CpdB-like	P08331	288	686	151	813	1301	152
	AYV64543	161	1579	162	581	607	153

^1^ The probes are shown in the same background color as Figure 1 and Table 1 to facilitate cross-referencing. * Numbers in parenthesis were obtained by removing the limit on type material in the TBlastN, which increased the number of genomes queried to 32,175. These four exceptional hits correspond to accession numbers CP080375.1, CP059263.1, CP051512.1, and CP074573.1.

**Table 15:** Specicity of probe O32133 for class *Bacilli*. Comparison between results obtained by querying type-material genomes of the class *Bacilli* and of the phylum *Bacillota* excluding *Bacilli*.

		Class *Bacilli* (Taxid:91061) (431 Type-Material Genomes)			Phylum *Bacillota* (Taxid:1239) Excluding Class *Bacilli* (Taxid:91061) (274 Type-Material Genomes)		
Type of Probe	Probe ^1^	Hits	Score Max.	Score Min.	Hits	Score Max.	Score Min.
UshA-like	P07024	90	235	151	30	230	151
	P44569	75	323	151	2	328	153
	WP_000726911	135	904	181	12	212	151
	WP_011837008	83	1297	151	62	439	154
	O32133	363	947	173	3	199	171
CpdB-like	P08331	231	686	151	57	521	152
	AYV64543	132	1579	162	54	509	156

^1^ The probes are shown in the same background color as Figure 1 and Table 1 to facilitate cross-referencing.

**Table 16:** *E. coli* complete genomes lacking either an *ushA*-like or a *cpdB*-like gene. These genomes represent a minor fraction (0.5%) of the total number of *E. coli* genomes in the NCBI Complete Genomes Database.

Accession	Description	*ushA*-like Score Max.	*cpdB*-like Score Max.
NZ_AP023205.1	*Escherichia coli* strain TUM18781 chromosome, complete genome	1100	88
NZ_CP128950.1	*Escherichia coli* strain TUM2805 chromosome, complete genome	1100	86
CP054239.1	*Escherichia coli* strain STO_Bone7 chromosome, complete genome	769	73
CP061232.1	*Escherichia coli* strain STEC639 chromosome, complete genome	678	71
AP027461.1	*Escherichia coli* str. K-12 substr. MG1655 D41c DNA, complete genome	87	1308
AP027460.1	*Escherichia coli* str. K-12 substr. MG1655 D37c16 DNA, complete genome	87	1308
AP027459.1	*Escherichia coli* str. K-12 substr. MG1655 D37c146 DNA, complete genome	87	1308
AP027458.1	*Escherichia coli* str. K-12 substr. MG1655 D37c145 DNA, complete genome	87	1308
AP027457.1	*Escherichia coli* str. K-12 substr. MG1655 D37c143 DNA, complete genome	87	1308
AP027456.1	*Escherichia coli* str. K-12 substr. MG1655 D37c13 DNA, complete genome	87	1308
AP027455.1	*Escherichia coli* str. K-12 substr. MG1655 D37b DNA, complete genome	87	1308
AP027454.1	*Escherichia coli* str. K-12 substr. MG1655 D33b DNA, complete genome	87	1308
AP027453.1	*Escherichia coli* str. K-12 substr. MG1655 D33a DNA, complete genome	87	1308
NC_011750.1	*Escherichia coli* IAI39, complete sequence	118	1307
CP042982.1	*Escherichia coli* strain NCCP 14540 chromosome, complete genome	88	1306
CP061269.1	*Escherichia coli* strain STEC1012 chromosome, complete genome	87	1305
CP099173.1	*Escherichia coli* strain RHB23-SO-C02 chromosome, complete genome	87	1303
NZ_AP027411.1	*Escherichia coli* strain EC521 isolate EC521 chromosome, complete genome	87	1300

**Table 17:** Avian pathogenic *E. coli* complete genomes containing an *ushA* and a *cpdB* gene, respectively, were found with probes P07024 and P08331.

Accession	Description	*ushA*-like Score Max.	*cpdB*-like Score Max.
NC_020163.1	*Escherichia coli* APEC O78, complete sequence	1102	1306
NZ_CP006834.2	*Escherichia coli* APEC O2-211 chromosome, complete genome	1098	1308
NZ_CP006830.1	*Escherichia coli* APEC O18 chromosome, complete genome	1097	1308
NZ_008563.1	*Escherichia coli* APEC O1, complete sequence	1097	1308
NZ_CP005930.1	*Escherichia coli* APEC IMT5155 chromosome, complete genome	592	1308

**Table 18:** *P. multocida* complete genomes lacking either an *ushA*-like or a *cpdB*-like gene. These genomes represent a minor fraction (3.6%) of the total number of *P. multocida* genomes in the NCBI Complete Genomes Database.

Accession	Description	*ushA*-like Score Max.	*cpdB*-like Score Max.
CP090521.1	*Pasteurella multocida* strain AH09 chromosome, complete genome	628	98
NZ_CP038871.1	*Pasteurella multocida* strain FCf15 chromosome, complete genome	151 *	847
NZ_CP084165.1	*Pasteurella multocida* strain s4 chromosome, complete genome	67	851
NZ_CP020345.1	*Pasteurella multocida* subsp. multocida strain CIRMBP-0884 chromosome, complete genome	67	851
NZ_CP113522.1	*Pasteurella multocida* strain PF13 chromosome, complete genome	67	851

* This score is the minimum required to compute the hit as an *ushA*-like gene. It is mentioned here because it is much lower than the immediately higher *ushA*-like score for this species (625; data for line 66 of Table S6).

## Data Availability

Data are contained within the article or supplementary material.

## Supplementary Materials

The following supporting information can be downloaded at: https://www.mdpi.com/article/10.3390/genes14081657/s1; Figure S1: Example of a launch page for a TBlastN search; Table S1: TBlastN analysis of *ushA*-like and *cpdB*-like genes in *Bacteria* phyla; Table S2: TBlastN analysis of *ushA*-like and *cpdB*-like genes in *Bacteria* classes from selected phyla; Table S3: TBlastN analysis of *ushA*-like and *cpdB*-like genes in *Bacteria* orders from selected classes; Table S4: TBlastN analysis of *ushA*-like and *cpdB*-like genes in *Bacteria* families from selected orders; Table S5: TBlastN analysis of *ushA*-like and *cpdB*-like genes in *Bacteria* genera from selected families; Table S6: TBlastN analysis of *ushA*-like and *cpdB*-like genes in selected *Bacteria* species; Table S7: Alphabetical lists of *Bacteria* taxa analyzed; Table S8: Alphabetical lists of taxa probed that contain no sequenced genomes in the NCBI Complete Genomes Database.
