# Supplementary figures and images for: Genomic Distribution of ushA-like Genes in Bacteria: Comparison to cpdB-like Genes
Source: Genes (Basel). 2023 Aug 20;14(8):1657. doi: 10.3390/genes14081657 (PMC10454023; doi:10.3390/genes14081657)

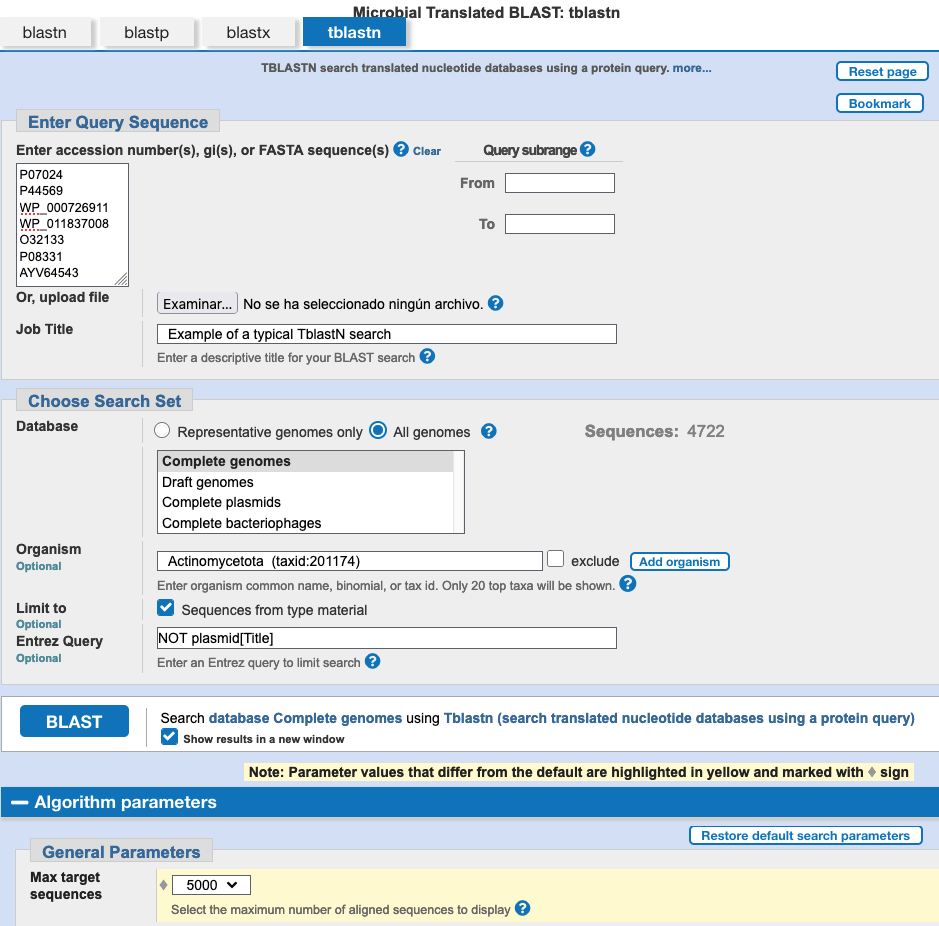

Supplement: Supplementary file 1 [file genes-14-01657-s001.zip › Figure S1.png]
